# Symbiont-mediated cytoplasmic incompatibility: What have we learned in 50 years?

**DOI:** 10.7554/eLife.61989

**Published:** 2020-09-25

**Authors:** J Dylan Shropshire, Brittany Leigh, Seth R Bordenstein

**Affiliations:** 1 Department of Biological Sciences, Vanderbilt UniversityNashvilleUnited States; 2 Vanderbilt Microbiome Initiative, Vanderbilt UniversityNashvilleUnited States; 3 Department of Pathology, Microbiology, and Immunology, Vanderbilt UniversityNashvilleUnited States; 4 Vanderbilt Institute for Infection, Immunology, and Inflammation, Vanderbilt University Medical CenterNashvilleUnited States; Max Planck Institute for Developmental BiologyGermany; Max Planck Institute for Developmental BiologyGermany

**Keywords:** cytoplasmic incompatibility, Wolbachia, prophage WO, CifA, CifB, endosymbiosis

## Abstract

Cytoplasmic incompatibility (CI) is the most common symbiont-induced reproductive manipulation. Specifically, symbiont-induced sperm modifications cause catastrophic mitotic defects in the fertilized embryo and ensuing lethality in crosses between symbiotic males and either aposymbiotic females or females harboring a different symbiont strain. However, if the female carries the same symbiont strain, then embryos develop properly, thereby imparting a relative fitness benefit to symbiont-transmitting mothers. Thus, CI drives maternally-transmitted bacteria to high frequencies in arthropods worldwide. In the past two decades, CI experienced a boom in interest due to its (i) deployment in worldwide efforts to curb mosquito-borne diseases, (ii) causation by bacteriophage genes, *cifA* and *cifB*, that modify sexual reproduction, and (iii) important impacts on arthropod speciation. This review serves as a gateway to experimental, conceptual, and quantitative themes of CI and outlines significant gaps in understanding CI’s mechanism that are ripe for investigation from diverse subdisciplines in the life sciences.

## Introduction

**Figure 1.**
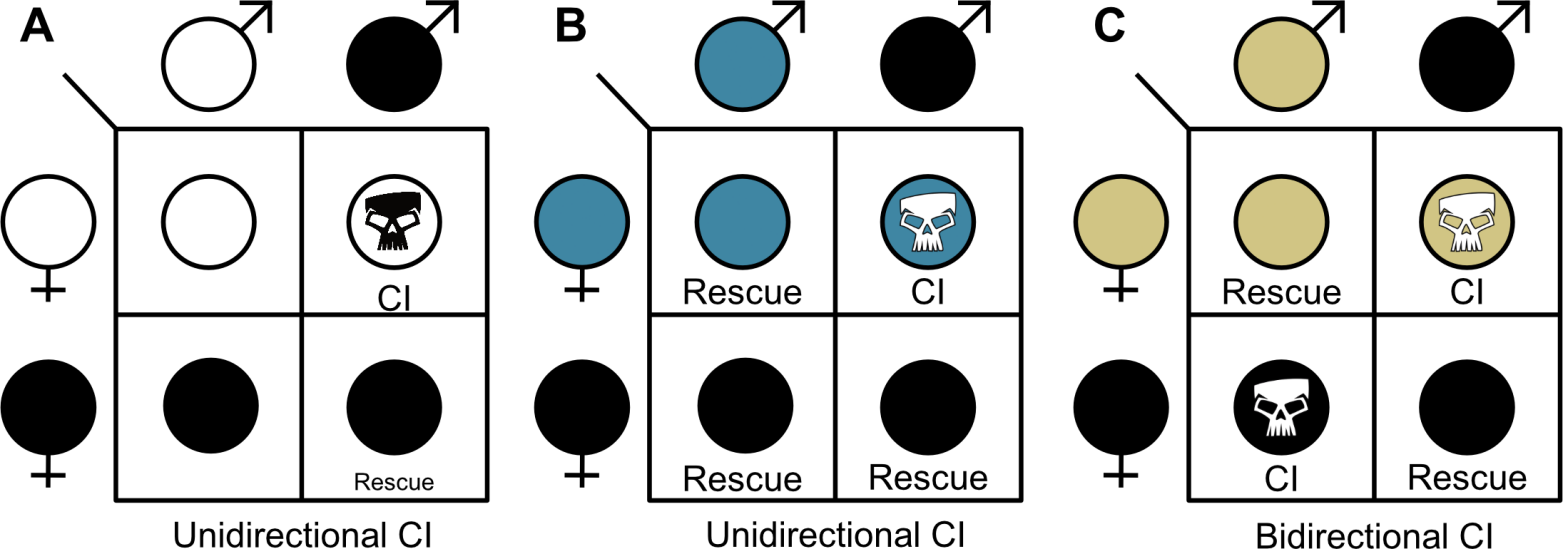
The three CI crossing relationships. (**A**) Unidirectional CI results in embryonic lethality when symbiont-containing males are crossed with aposymbiotic females. Rescue of this embryonic lethality occurs if the female carries a compatible symbiont strain. (**B**) In some cases, unidirectional CI can emerge when one strain can rescue another strain, but the other strain does not reciprocate the rescue. (**C**) Bidirectional CI occurs when incompatible strains are present in a population. Rescue occurs if the female likewise harbors the same strain. Filled sex symbols indicate symbiotic hosts. Different colors represent different symbiont strains. Skull symbols represent embryonic death.

From 1938 through the 1960s, an enigmatic, intraspecific incompatibility that caused embryonic death was reported between geographically isolated strains of *Culex pipiens* mosquitoes (Laven, 1951; Marshall, 1938), *Aedes scutellaris* mosquitoes (Smith-White and Woodhill, 1955), and *Nasonia vitripennis* parasitoid wasps (Ryan and Saul, 1968). Crossing experiments in both *Culex* and *Nasonia* surprisingly revealed that the incompatibility was caused by a maternally-inherited cytoplasmic factor (Laven, 1951; Ryan and Saul, 1968). This cytoplasmic incompatibility (CI) manifested as embryonic death when males carried the factor, but it was rescued if the female was from the same maternal lineage (Figure 1A). Intriguingly, *Cu. pipiens* (Laven, 1951) and *N. vitripennis* (Ryan and Saul, 1968) that had this cytoplasmic factor were either compatible, unidirectionally incompatible (Figure 1A,B), or bidirectionally incompatible (Figure 1C) with strains of different geographic origin. The underlying cause of these incompatibilities would remain a mystery for several decades.

Motivated by the finding that Typhus is a Rickettsial disease (da Rocha-Lima, 1916), microbiologists Hertig and Wolbach conducted a survey of *Rickettsia*-like bacteria among numerous arthropod orders in and around Boston, Massachusetts in 1924 (Hertig and Wolbach, 1924). The bacteria were classified as *Rickettsia*-like based on size (often smaller than other bacteria), shape (cocciform or rod), Gram staining (gram-negative), and a Giemsa nucleotide stain (to separate microscopy artifacts from cells with DNA). In the *Cu. pipiens* mosquito, they found tiny rod-like or coccoid, gram-negative, Rickettsial bacteria residing within male and female reproductive cells (Hertig and Wolbach, 1924). The bacteria were absent in various other tissues including oesophageal diverticula, gut tissues, malphighian tubes, fat-body, heart and pericardial cells, salivary glands, and accessory reproductive organs (Hertig and Wolbach, 1924). Intriguingly, when *Cu. pipiens* with the bacteria were reared in the lab, offspring also harbored them in their reproductive tissues as early larva, suggesting that the bacteria were maternally inherited (Hertig and Wolbach, 1924). These bacteria would later be named *Wolbachia pipientis* by Dr. Marshall Hertig: *Wolbachia* for Dr. Simeon Burt Wolbach, Hertig’s PhD advisor, and *pipientis* for the mosquito it was discovered in Hertig, 1936. In this review, we will refer to the symbiont as *Wolbachia* since it currently remains a genus of only one recognized species.

In 1971, Yen and Barr investigated the effects of CI on embryonic development and discovered *Rickettsia*-like bacteria matching the description of *Wolbachia* in the eggs of symbiont-bearing *Cu. pipiens* females (Yen and Barr, 1971). This finding led them to the breakthrough hypothesis that CI is caused by these long-overlooked bacteria (Yen and Barr, 1971). They later tested this hypothesis using crosses with antibiotic-treated and untreated *Cu. pipiens* mosquitoes and determined that CI is a symbiont-derived phenotype caused by *Wolbachia* (Yen and Barr, 1973), thus substantiating *Wolbachia* as the etiological agent of CI phenotypes. This initial characterization of *Wolbachia* and CI in *Cu. pipiens* opened the floodgates, with many ensuing studies reporting cases of CI-inducing *Wolbachia* in Diptera (Baton et al., 2013; Bian et al., 2013; Hoffmann et al., 1986; Riegler and Stauffer, 2002), Hymenoptera (Betelman et al., 2017; Dittmer et al., 2016), Coleoptera (Kajtoch and Kotásková, 2018), Hemiptera (Ju et al., 2017; Ramírez-Puebla et al., 2016), Orthoptera (Martínez-Rodríguez and Bella, 2018), Lepidoptera (Arai et al., 2019; Hornett et al., 2008), Thysanoptera (Nguyen et al., 2017), Acari (Gotoh et al., 2007; Gotoh et al., 2003; Vala et al., 2002), Isopoda (Cordaux et al., 2012; Sicard et al., 2014), and Arachnids (Curry et al., 2015).

Among these orders, *Wolbachia* are highly diverse and phylogenetically divided into 17 ‘supergroups’ (denoted A-S, excluding G and R), and CI-inducing *Wolbachia* are so far restricted to supergroups A and B (Lefoulon et al., 2020; Lo et al., 2007; Wang et al., 2016). However, despite the considerable diversity between *Wolbachia* strains, the most studied models for CI are the *Wolbachia* of *Culex* (*w*Pip), *Drosophila* (*w*Ri and *w*Mel), *Nasonia* (*w*VitA and *w*VitB), and *Laodelphax* (*w*Str). Aside from *Wolbachia*, the far less common (Weinert et al., 2015; Zchori-Fein and Perlman, 2004) Bacteroidetes bacteria *Cardinium* were found to cause CI nearly three decades later (Hunter et al., 2003; Yen and Barr, 1973). Additionally, unknown symbionts of *Brontispa longissimi* coconut beetles and *Lariophagus distinguendus* parasitoid wasps cause CI, but they are not *Wolbachia* or *Cardinium* (König et al., 2019; Takano et al., 2017). In addition, Gammaproteobacteria of the genus *Rickettsiella* cause CI in *Mermessus fradeorum* spiders (Rosenwald et al., 2020). This review will focus primarily on *Wolbachia*-induced CI, but other symbionts will be discussed when information is available.

**Figure 2.**
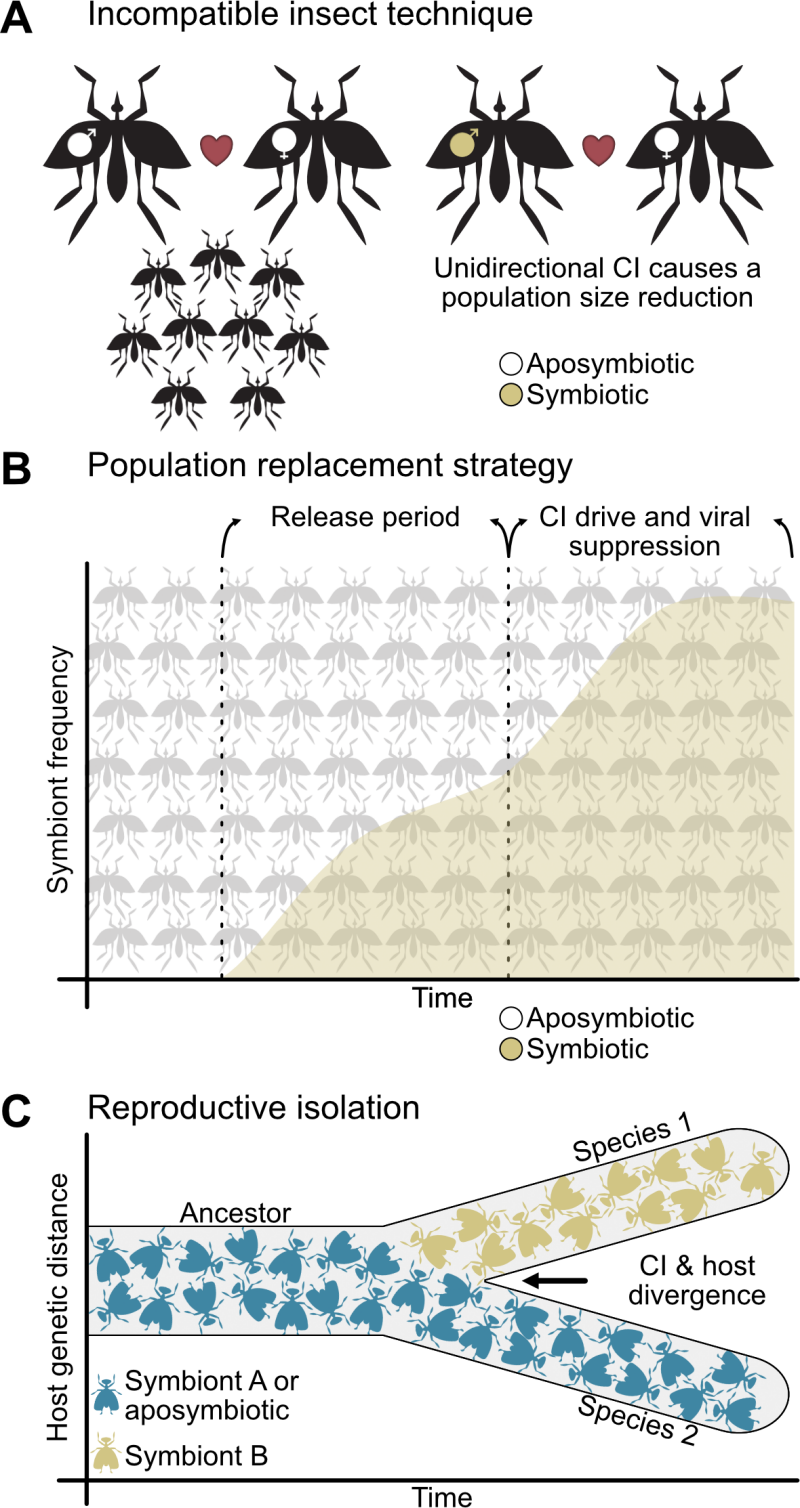
CI is important to vector control and reproductive isolation between species. (**A**) The incompatible insect technique is used to reduce population sizes (Crawford et al., 2020; Laven, 1967). Typically, two aposymbiotic individuals will mate and produce viable offspring (left), but if males bearing CI-inducing symbionts are released into the population, then they will cause unidirectional CI when they mate with aposymbiotic females (right) or bidirectional CI when they mate with females harboring incompatible symbionts (not shown). This yields a reduction in egg hatching and population size. (**B**) The population replacement strategy involves the release of both males and females bearing CI-inducing and pathogen blocking symbionts (Hoffmann et al., 2011; O'Neill, 2018). After a period of releases, CI will spread the symbiont to high frequencies where it can block the replication of human diseases. (**C**) CI-inducing symbionts can cause reproductive isolation through unidirectional or bidirectional CI when different individuals, populations, or species have different incompatible symbiont states (Bordenstein et al., 2001; Breeuwer and Werren, 1990; Gebiola et al., 2017; Jaenike et al., 2006). This reproductive barrier reduces gene flow between hosts with different symbiont states, allowing for their divergence.

CI has attracted considerable, applied interest in the last decade from scientists, companies, and governments because it is at forefront of efforts to reduce the spread of dengue, Zika, and other arboviral infections (Caragata et al., 2016; Crawford et al., 2020; Ford et al., 2019; Hoffmann et al., 2011; O'Connor et al., 2012; O'Neill, 2018; Rasgon, 2007; Rasgon, 2008; Teixeira et al., 2008; WHO, 2016; Xi et al., 2005). Two CI-based vector control strategies are deployed worldwide. First, the incompatible insect technique (IIT), also known as population suppression, aims to reduce the population size of disease vectors through release of CI-inducing male insects (Figure 2A; Ant et al., 2020; Caputo et al., 2020; Chambers et al., 2011; Crawford et al., 2020; Fresno, 2018; Kyritsis et al., 2019; Laven, 1967; Mains et al., 2019; Mains et al., 2016; O'Connor et al., 2012; Puggioli et al., 2016; Zheng et al., 2019a). Conversely, the population replacement strategy (PRS) does not reduce population sizes, but instead it aims to convert a native population that transmits arboviruses to humans with one that has reduced vectoral capacity (Figure 2B; Caragata et al., 2016; Hoffmann et al., 2011; Moreira et al., 2009; O'Neill, 2018; Tantowijoyo et al., 2020; Teixeira et al., 2008; van den Hurk et al., 2012). PRS uses two characteristics of CI-*Wolbachia*: the ability to rapidly spread through populations using CI and the ability of some strains to inhibit replication of arboviruses including dengue, Zika, chikungunya, and yellow fever (Caragata et al., 2016; Moreira et al., 2009; Teixeira et al., 2008; van den Hurk et al., 2012). When male and female mosquitoes bearing pathogen blocking *Wolbachia* are released to sufficiently high frequencies, CI drives them to frequencies approaching fixation that significantly alleviates the transmission of disease in the region. Both methods have been widely successful in their respective approaches (Crawford et al., 2020; Fresno, 2018; O'Neill et al., 2018; Tantowijoyo et al., 2020).

In addition to combatting arthropod-borne diseases, CI has attracted interest from evolutionary biologists because it can cause reproductive isolation and thus be a contributor to speciation (Figure 2C). The Biological Species Concept defines groups of individuals as different species if they cannot interbreed (Dobzhansky, 1937; Mayr, 1963), and species emerge when reproductive isolation between two populations prevents gene flow (Coyne, 2001). Bidirectional and unidirectional CI can both reproductively isolate populations with different symbiont states, but to different degrees (Brucker and Bordenstein, 2012). Since bidirectional CI restricts gene flow in both cross directions, it can strongly reproductively isolate populations that harbor incompatible symbionts. This is indeed the case between *Nasonia* parasitoid wasps that diverged between ~0.25 and 1 million years ago (Bordenstein et al., 2001; Breeuwer and Werren, 1990). Alternatively, unidirectional CI restricts gene flow in one direction and does not appear to contribute to speciation in some host-*Wolbachia* symbioses such as in the *D. yakuba* clade (Cooper et al., 2017). However, North American populations of male symbiont-bearing *D. recens* cause unidirectional CI when mated with aposymbiotic *D. subquinaria* (Jaenike et al., 2006; Shoemaker et al., 1999), reducing gene flow between populations. Intriguingly, *Cardinium* yields similar patterns of asymmetric unidirectional CI between lab populations of *Encarsia suzannae* and its sister species *E. gennaroi* (Gebiola et al., 2017). Together, these studies suggest a role for symbiont-induced CI in reproductive isolation and incipient speciation.

In this review, we comprehensively synthesize the CI literature by discussing the rapid advances in understanding CI’s genetic basis, biochemical properties associated with CI, CI-associated abnormalities, CI strength variation, and host factors that correlate with CI expression. The reviewed works provide a concrete foundation for new testable and robust models, hypotheses, and evidence. Thus, we end with a description of the field’s models to explain the mechanistic underpinnings of CI and place them into the framework of current literature. Under these models, we identify key predictions and questions that motivate future areas of research to continue to build textbook knowledge on one of the most widespread selfish adaptations of symbionts.

## What is the genetic basis of CI?

### Identifying the CI and rescue genes

Until the past decade, the genetic basis of CI remained elusive. The intangibility of the CI genes was due in no small part to the inability to genetically engineer symbionts such as *Wolbachia* (Iturbe-Ormaetxe et al., 2007; Thiem, 2014). Progress in CI genetics became possible with the genome sequencing of the *w*Mel *Wolbachia* of *D. melanogaster* in 2004 (Wu et al., 2004). *w*Mel has a streamlined genome with numerous mobile elements including phages (Wu et al., 2004). Notable among these elements was *Wolbachia*’s phage WO, which was first identified in 1978 via transmission electron microcopy of viral-like particles in symbiont-bearing *Cu. pipiens* (Wright et al., 1978). Prophage WO encodes a set of proteins termed the Eukaryotic Association Module that share homology to eukaryotic proteins, likely due to lateral gene transfer from eukaryotes to the phage, and they are predicted to interact with eukaryotic processes (Bordenstein and Bordenstein, 2016). *w*Mel’s genome is also enriched with ankyrin proteins that are involved in protein-protein interactions in eukaryotes and are relatively rare in free living bacteria as compared to intracellular bacteria and eukaryotes (Al-Khodor et al., 2010; Jernigan and Bordenstein, 2014). Conversely, genome sequencing of the mutualistic *w*Bm *Wolbachia* of *Brugia malayia* nematodes revealed it did not contain phage WO nor an enrichment of ankyrins (Foster et al., 2005). These findings suggested a correlation between reproductive parasitism and the presence of phage WO and/or ankyrin genes and motivated hypotheses that phage WO may be involved in CI (Foster et al., 2005; Wu et al., 2004; Yamada et al., 2011). Numerous additional genome sequencing projects would also be integral to identifying candidate genes for CI and rescue, including *w*Pip, *w*Au, *w*Ri, *w*Ha, and *w*Rec (Klasson et al., 2009; Klasson et al., 2008; Metcalf et al., 2014; Salzberg et al., 2009; Sutton et al., 2014).

The first attempt to functionally dissect CI’s genetic basis generated a list of 12 gene candidates in the *w*Mel genome based on putative host interaction: nine ankyrin genes (WD0294, WD0385, WD0498, WD0514, WD0550, WD0633, WD0636, WD0754, and WD0776), two virulence-related genes (WD0579 and WD0580), and one phage-associated methylase gene (WD0594) (Yamada et al., 2011). Since *Wolbachia* are not genetically tractable (Iturbe-Ormaetxe et al., 2007; Thiem, 2014), *D. melanogaster* transgenic tools were used to test these gene candidates (Duffy, 2002). However, transgenic expression of these genes in aposymbiotic male flies revealed that none recapitulated CI (Yamada et al., 2011). Moreover, neither transcriptional nor genetic variation of *Wolbachia*’s ankyrin genes correlated with a strain’s ability to induce CI (Duron et al., 2007b; Papafotiou et al., 2011).

Additional ‘omic studies would pave the way for identification of new gene candidates. First, mass spectrometry and SDS-page analyses of spermatheca (the female sperm storage organ) extracts from symbiont-bearing *Cu. pipiens* females revealed the prophage WO protein WPIP0282 (Beckmann and Fallon, 2013), thus elevating a new candidate for CI and/or rescue and providing additional support to hypotheses that phage WO genes may contribute to CI. Second, genomic comparisons of the *w*Mel genome against the genome of the non-parasitic *w*Au strain of *D. simulans* revealed nine genes absent in the non-parasitic strain that were present in *w*Mel (Sutton et al., 2014). These candidates included numerous genes from *Wolbachia*’s prophage WO including WD0631 that is a *w*Mel homolog of *w*Pip’s WPIP0282, WD0632 which is adjacent to WD0631, and a set of transcriptional regulators (Sutton et al., 2014). Thus, evidence continued to build around phage WO genes as CI factors. Finally, sequencing of the *w*Rec genome revealed a highly reduced prophage WO with approximately one-quarter the number of genes in a close relative (Metcalf et al., 2014). These genes contained several previously described candidates including the *w*Rec homolog of WPIP0282, four transcriptional regulators also absent in *w*Au, and a *Wolbachia* transcriptional regulator gene *wtrM* linked to regulation of host meiosis in *Cu. molestus* (Beckmann and Fallon, 2013; Pinto et al., 2013; Sutton et al., 2014).

The reduced *w*Rec genome would later form the basis of an unbiased, comparative ‘omic study assessing the genomes of CI-inducing *Wolbachia*, a genome of a non-parasitic strain and a transcriptome and proteome of *Wolbachia*-carrying ovaries (LePage et al., 2017). This analysis revealed only two phage WO genes in the Eukaryotic Association Module, WD0631 and the adjacent WD0632, as CI candidate genes in the *w*Mel strain of *D. melanogaster* (LePage et al., 2017). These genes would also later be determined to be absent in the parthenogenesis-inducing *w*Tpre strain of *Trichogramma* wasps (Lindsey et al., 2018). WD0631 and WD0632 were named *cytoplasmic incompatibility factors A* and *B* (*cifA* and *cifB*), respectively (LePage et al., 2017). The gene is referred to in lowercase and italics (*cifA* and *cifB*), the protein is referred to in uppercase with no italics (CifA and CifB), and the strain that the specific *cif* gene comes from can be defined with the strain name as a subscript (*cif_wMel_* or *cif_wPip_*). This gene nomenclature is consistent with guidelines from the American Society for Microbiology ('The Journal of Bacteriology, 2018').

**Figure 3.**
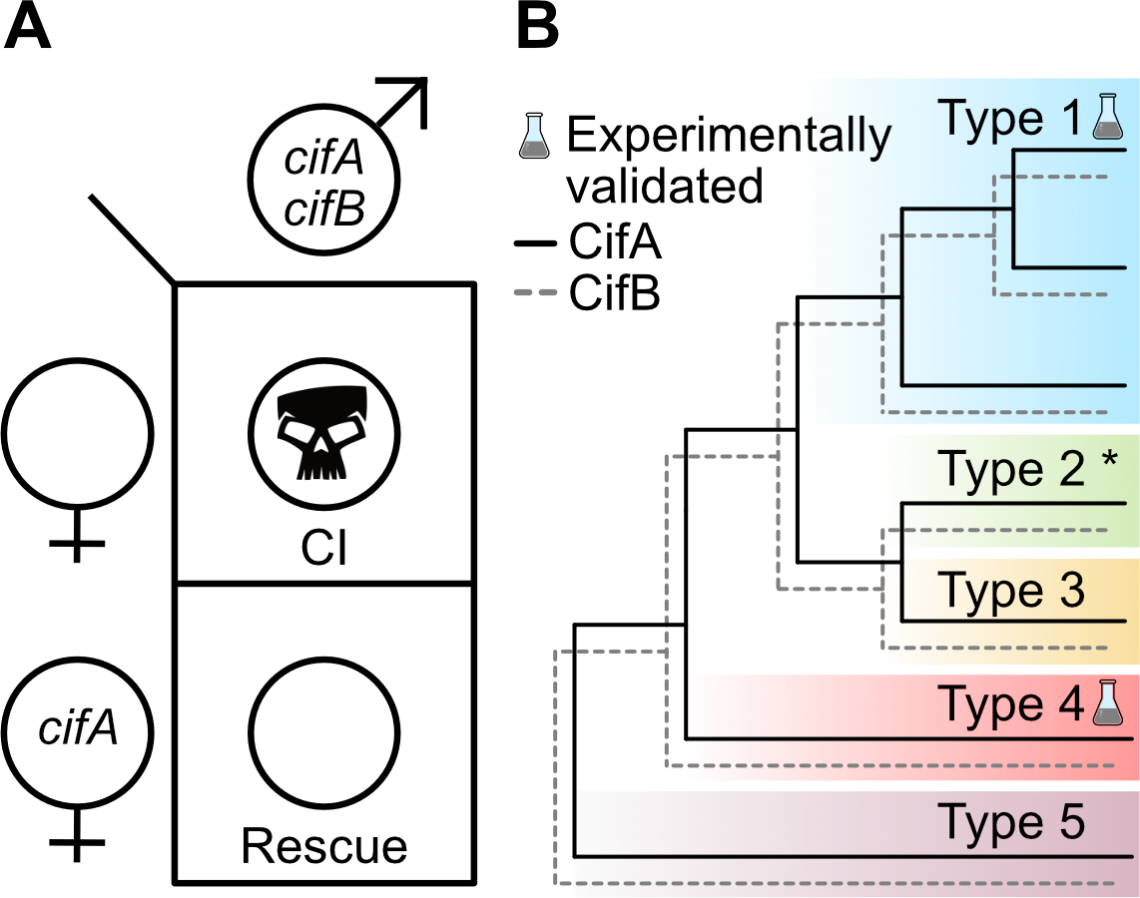
Two-by-One genetic model of *cif*-induced CI and Cif phylogeny. (**A**) The Two-by-One genetic model of CI surmises that both c*ifA* and c*ifB* must be expressed in males to cause CI, and c*ifA* must be expressed in females to rescue CI (Shropshire and Bordenstein, 2019). (**B**) CifA and CifB codiverge and are classified into at least five different phylogenetic Types (1-5) (Bing et al., 2020b; LePage et al., 2017; Lindsey et al., 2018; Martinez et al., 2020). To date, only Type 1 *cifs* from *w*Mel and *w*Pip, and Type 4 *cifs* from *w*Pip, have been experimentally confirmed to cause and rescue CI (Beckmann et al., 2017; Chen et al., 2019; LePage et al., 2017; Shropshire et al., 2018; Shropshire and Bordenstein, 2019). Moreover, unpublished results from JDS and SRB suggest that Type 2 *cifs* from *w*Ri are CI and rescue-capable (denoted with an asterisk).

With independent ‘omic identification of *cifA* and *cifB* as candidates for CI (Beckmann and Fallon, 2013; LePage et al., 2017; Sutton et al., 2014), two studies simultaneously explored the relationship between *cif_wMel_* (LePage et al., 2017) and *cif_wPip_* (Beckmann et al., 2017) genes and CI using transgenic expression systems in *D. melanogaster*. Singly expressing *cifA_wMel_* or *cifB_wMel_* in aposymbiotic *D. melanogaster* males failed to induce CI, but dual expression of the genes caused rescuable CI-like hatch rates and cytological embryonic defects (LePage et al., 2017), suggesting that the *cif_wMel_* genes cause CI only when expressed together. Similar results were reported when *cifA_wPip_* and *cifB_wPip_* were dually expressed in aposymbiotic *D. melanogaster* males, but rescue was not achieved (Beckmann et al., 2017), indicating that some biological or technical limitation of the system may have inhibited the ability to rescue transgenic *cif_wPip_* CI in a heterologous expression system. Later, similar transgenic experiments revealed that *cifA_wMel_* expression in aposymbiotic *D. melanogaster* females can rescue CI (Shropshire et al., 2018), motivating a Two-by-One genetic model of CI wherein *cifA* and *cifB* cause CI unless *cifA* is expressed in the ovaries or embryo to rescue it (Figure 3A). This model was further supported through transgenic expression of *cifA_wMel_* and *cifB_wMel_* in aposymbiotic males to induce transgenic CI and through crossing them to *cifA_wMel_*-expressing aposymbiotic females to show that transgenic CI can be rescued at levels comparable to symbiont-bearing females (Shropshire and Bordenstein, 2019).

Notably, while the Two-by-One genetic model is most consistent with transgenic expression studies in *D. melanogaster* that achieve rescuable CI (Beckmann et al., 2017; Chen et al., 2019; LePage et al., 2017; Shropshire et al., 2018; Shropshire and Bordenstein, 2019), transgenic expression of *cifB_wMel_* and *cifB_wPip_* in yeast can cause temperature sensitive lethality that can be inhibited by co-expression with cognate *cifA* (Beckmann et al., 2017). Although since yeast do not have sperm or eggs, which are the targets of CI, the relevance of phenotypes observed in a heterologous yeast expression system need to be replicated in insect models. While a divergent *cifB* gene variant from *w*Pip can weakly reduce embryonic hatching when crossed to aposymbiotic females (Chen et al., 2019), it remains unknown if this lethality can be rescued. These data lend support to the possibility that some strains may employ a model of CI wherein *cifB* is the CI-causing factor and *cifA* is only the rescue factor. However, interpretation of these results is significantly complicated by the absence of rescue data for any *cifB*-associated reduction in embryonic hatching in insects. Moreover, mutagenesis analyses, described in further detail below, indicate that changing conserved residues across CifA can crucially prevent CI, lending additional support for CifA’s important role as a CI-inducing factor (Shropshire et al., 2020). More functional genetic analyses will be necessary to provide evidence for an alternative to the Two-by-One genetic model of CI. Hereafter, we will discuss relevant phenomena in the context of a Two-by-One genetic model.

### Phylogenetics of the *cifA* and *cifB* genes that cause and rescue CI

Initial comparative sequence analysis of Cif proteins revealed that CifA and CifB have concordant phylogenies with considerable divergence across several distinct phylogenetic clades (LePage et al., 2017). Since then, the availability of additional genomes and Cif sequences have exposed at least five clades referred to as Types 1–5 (Figure 3B; Bing et al., 2020b; Lindsey et al., 2018; Martinez et al., 2020), and highly divergent Cif-like homologs in *Orientia* and *Rickettsia* bacteria (Gillespie et al., 2018). It is likely that continued genomic sequencing will reveal additional Cif phylogenetic Types. The *w*Mel Cif proteins belong to the Type 1 clade, and *w*Pip has both Type 1 and Type 4 Cif proteins. The phylogenetic classification of a *cif* gene can be indicated with a T# subscript in brackets to the right of the gene name (i.e. *cif_wMel[T1]_* or *cif_wPip[T4]_*).

While the *cif* genes are associated within the Eukaryotic Association Module of prophage WO or WO-like islands (Bordenstein and Bordenstein, 2016), Cif phylogeny is not concordant with phage WO or *Wolbachia* phylogeny, potentially reflecting the typically high rates of inter- and intragenic recombination in phage genomes (Bordenstein and Wernegreen, 2004; LePage et al., 2017). Some *cif* genes are flanked by ISWpi1 transposons which may assist horizontal transfer between WO-associated regions or *Wolbachia* strains, but it remains unclear if they alone are responsible for divergence between the phylogeny of the *cif* genes, *Wolbachia*, and phage WO (Cooper et al., 2019; Madhav et al., 2020). To date, only *cif* genes belonging to the Type 1 and 4 clades have been experimentally evaluated and confirmed to cause and rescue CI (Beckmann et al., 2017; Chen et al., 2019; LePage et al., 2017; Shropshire et al., 2018; Shropshire and Bordenstein, 2019), but unpublished data suggest *cif* genes in the Type 2 clade can cause and rescue CI (JDS and SRB, unpublished data). These studies indicate that despite considerable sequence divergence, proteins across the phylogenetic landscape of the Cifs remain capable of causing and rescuing CI. Though, the phenotypic output of the Type 3, Type 5, and *Orientia*/*Rickettsia cif*-like genes have not been experimentally assessed, and it remains unknown if they can contribute to CI phenotypes. However, the CI-inducing strains *w*No of *D. simulans* and *w*Stri of *La. striatellus* only have Type 3 or Type 5 genes, respectively (Bing et al., 2020b; LePage et al., 2017), suggesting these genes may cause CI and rescue. Since CI has not been reported in *Orientia* or *Rickettsia* species, it is less likely these distant *cif*-like homologs contribute to CI (Gillespie et al., 2018). Alternatively, other yet identified gene sets may be CI-capable in CI-causing symbionts. Indeed, genomic and transcriptomic sequencing of CI-inducing *Cardinium* reveals that they do not carry obvious homologs to the CI genes (Mann et al., 2017). More functional genetic studies will be necessary to identify and assess the landscape of *cif*-like genes and to identify alternative CI genes.

### Relationships between *cif* sequence diversity and CI phenotypes

Theory predicts that selection should favor the maintenance of rescue, but not CI, when *Wolbachia* are at equilibrium in the population since CI would no longer increase *Wolbachia*’s prevalence in the population (Turelli, 1994). In this context, and under the Two-by-One genetic model of CI in which *cifA* is involved in both CI and rescue while *cifB* is involved in CI, putative loss-of-function mutations in CI will accumulate preferentially in *cifB*, though not universally, relative to *cifA*. Consistent with this hypothesis, there are approximately twice as many putative loss-of-function mutations in *cifB* relative to *cifA* (Martinez et al., 2020), suggesting that CI is ablated more frequently than rescue. For example, the *w*PanMK and *w*PanCI *Wolbachia* strains of *D. pandora* cause male and CI respectively, but the male-killing strain has an early stop codon in CifB that putatively inhibits function and may allow for the phenotypic switch from CI to male (Asselin et al., 2018). Additionally, the *w*Mau *Wolbachia* of *D. mauritiana* encodes Type 3 Cif proteins (LePage et al., 2017; Meany et al., 2019) and does not cause CI, but it can rescue CI caused by the closely related *w*No of *D. simulans* (Bourtzis et al., 1998; Rousset and Solignac, 1995; Zabalou et al., 2008). CifA*_w_*_Mau[T3]_ sequence is identical to the CI and rescue-capable *w*No *Wolbachia* strain, but CifB*_w_*_Mau[T3]_ has a frameshift that introduces over ten stop codons that in turn associates with the loss of CI (Meany et al., 2019). However, it is important to note that two CI-capable *Wolbachia*, *w*Yak of *D. yakuba* and *w*Rec of *D. recens* (Cooper et al., 2017; Shoemaker et al., 1999), have putative loss-of-function mutations in the form of truncations in all of their *cifB* genes (Martinez et al., 2020). These results suggest that while the homologs appear pseudogene-like, they may be functional. Indeed, dual expression of *cifA;B_wRec[T1]_* transgenes in aposymbiotic males yields a rescuable hatch rate reduction (JDS and SRB, unpublished data). In sum, loss-of-function mutations are common in *cifB*, resulting in ablation of CI while maintaining rescue. Moreover, evolution-guided mutagenesis assays across the Cif proteins reveal that conserved residues in the CifA C-terminal domain of unknown function are crucial for CI (Shropshire et al., 2020). Thus, it is plausible that seemingly innocuous amino acid changes within this CifA region may ablate CI and yet maintain rescue. Higher resolution comparative genomic analyses coupled with phenotypic data will be necessary to evaluate this hypothesis. More work will also be necessary to functionally assess the impacts of putative loss-of-function mutations on CI and rescue capabilities.

While the above examples clearly highlight a relationship between *cif* sequence variation and loss of CI, there are other strains that are more difficult to explain. For example, *w*Suz of *D. suzukii* encodes both Type 1 and Type 2 *cif* genes that are highly similar to the strong CI-inducing strain of *w*Ri, but *w*Suz does not cause CI (Cattel et al., 2018; Conner et al., 2017; Hamm et al., 2014; Lindsey et al., 2018). While CifA*_w_*_Suz[T2]_ has been disrupted by the insertion of a transposase, the Cif*_w_*_Suz[T1]_ gene pair remains intact and has only 2–4 amino acid substitutions relative to Cif*_w_*_Ri[T1]_ (Conner et al., 2017; Lindsey et al., 2018). In theory, the Type 1 gene set alone should be CI-capable (Lindsey et al., 2018); though Cif*_w_*_Suz[T1]_ mutations may be in key residues for CI expression. Notably, *w*Ri does not cause CI when transinfected into *D. suzukii* (Cattel et al., 2018). Thus, the Cif*_w_*_Suz[T1]_ proteins may be able to cause CI, but their effects are inhibited by suppressors encoded in the *D. suzukii* genome. In a separate example, the triple-strain infection of *w*AlbA, *w*AlbB, and *w*Mel in *A. albopictus* can cause CI but cannot self-rescue (Ant and Sinkins, 2018). Since each of the individual *Wolbachia* strains in this triple-strain infection can cause CI and are self-compatible in *A. aegypti* (Ant and Sinkins, 2018), neither genetic variation in the *cif* genes alone nor host suppressors can explain the emergence of self-incompatibility. However, it is plausible that some *Wolbachia* may inhibit the reproductive manipulations of other co-infecting strains, but this requires further testing. In summary, the relationships between *Wolbachia* strains and their hosts are likely to have an impact on CI. Additional work is necessary to answer these persistent questions: how does superinfection impact CI expression, how does the host act to suppress CI phenotypes, and what are the evolutionary dynamics that govern these interactions?

### The genetic basis of bidirectional CI remains unknown

There is strong evidence for the genetic basis of unidirectional CI between *Wolbachia*-bearing and aposymbiotic arthropods (Figure 1A; Beckmann et al., 2017; Chen et al., 2019; LePage et al., 2017; Shropshire et al., 2018; Shropshire and Bordenstein, 2019). However, the genetic basis of bidirectional CI between arthropods with different symbiont strains remains poorly understood (Figure 1B,C). Sequence divergence in CI-associated factors has long been thought to be a contributing factor to these incompatibilities, namely that divergence in both CI-causing and rescue-causing genes would be required for bidirectional CI to emerge (Charlat et al., 2001). Indeed, phylogenetic analyses of *cif* genes reveal that strains carrying similar alleles tend to be compatible, strains with more distantly related *cif* genes are not, and a single *Wolbachia* strain can have multiple unique *cif* gene pairs (Bonneau et al., 2019; Bonneau et al., 2018a; LePage et al., 2017). For instance, when *w*Mel is transinfected into a *D. simulans* background, it is unidirectionally incompatible with the native *w*Ri strain, wherein *w*Ri can rescue *w*Mel-induced CI but the reciprocal cross is incompatible (Poinsot et al., 1998). Intriguingly, *w*Ri carries Type 1 *cif* genes closely related to *w*Mel’s and a divergent Type 2 gene pair. Thus, it is plausible that *w*Ri can rescue *w*Mel’s CI because of CifA*_w_*_Ri[T1],_ whereas *w*Mel cannot rescue *w*Ri’s CI because it lacks a rescue gene for the Type 2 gene pair (LePage et al., 2017). Additionally, population genetic analyses of *cif* genes in *w*Pip reveal that there are numerous unique strains, each strain carries multiple closely-related *cif* variants that belong to Type 1 and Type 4 *cif* clades, and a single genetic variant of CifB*_w_*_Pip[T1]_ correlates with the inability of one strain of *w*Pip to rescue CI caused by a divergent *w*Pip strain (Atyame et al., 2011b; Bonneau et al., 2019; Bonneau et al., 2018a). However, while these data suggest that *cif* genetic variation and/or copy number contributes to strain incompatibility, it remains possible that the considerable host genotypic variation between these incompatible populations contributes to these relationships in a way that also correlates with *cif* genotypic diversity (Atyame et al., 2011a). More reductionist functional studies that control for variation in host genotype will be necessary to confirm that *cif* sequence variation alone can explain CI relationships.

Historically, CI and rescue were thought to be caused by different genes, and that divergence in both genes would be required for bidirectional CI to evolve relative to an ancestral strain (Charlat et al., 2001). Thus, this model for bidirectional CI requires two steps: one mutation for CI and one for rescue. A major limitation of this model is that the intermediate state, wherein only one of the two phenotypes have shifted, is self-incompatible and represents a ‘maladaptive valley’ unlikely to persist as a rare variant. Given the abundance of bidirectionally incompatible strains across the arthropod *Wolbachia* (Atyame et al., 2011b; Bordenstein and Werren, 2007; Branca et al., 2009; O'Neill and Karr, 1990; Sicard et al., 2014), and the rarity of so-called ‘suicidal’ self-incompatible strains (Zabalou et al., 2008), crossing this maladaptive valley may be an unlikely evolutionary scenario. In contrast, since CifA is involved in both CI and rescue, it becomes possible for a single mutation that affects CI to also impact rescue. Thus, a single mutation in CifA may shift both CI and rescue phenotypes, yield bidirectional CI relative to an ancestor, and maintain self-compatibility (Shropshire et al., 2018). Indeed, mutagenesis of highly conserved amino acids across CifA reveal that sites within CifA’s N-terminal region are crucially important for the expression of both CI and rescue, suggesting that residues in this single region are coopted for both phenotypes (Shropshire et al., 2020). Notably while this one-step model of bidirectional CI avoids the maladaptive valley, it may only spread if transferred into a new aposymbiotic (sub)population since emergence of a new incompatibility type within a symbiont-bearing population would be immediately incompatible with the more common symbiont in the population. More research is needed to fully understand the genetic basis of bidirectional CI and its evolution. For instance, theoretical modeling will be necessary to evaluate additional routes of emergence as it relates to Cif sequence variation, functional genetic assays can be used to unravel the correlation between *cif* sequence variation and (in)compatibility relationships, and population genetic surveys coupled with population dynamics modeling would reveal when a novel variant would be likely to persist in a population.

## What is the mechanistic basis of Cif-induced CI?

### CifA molecular function

**Figure 4.**
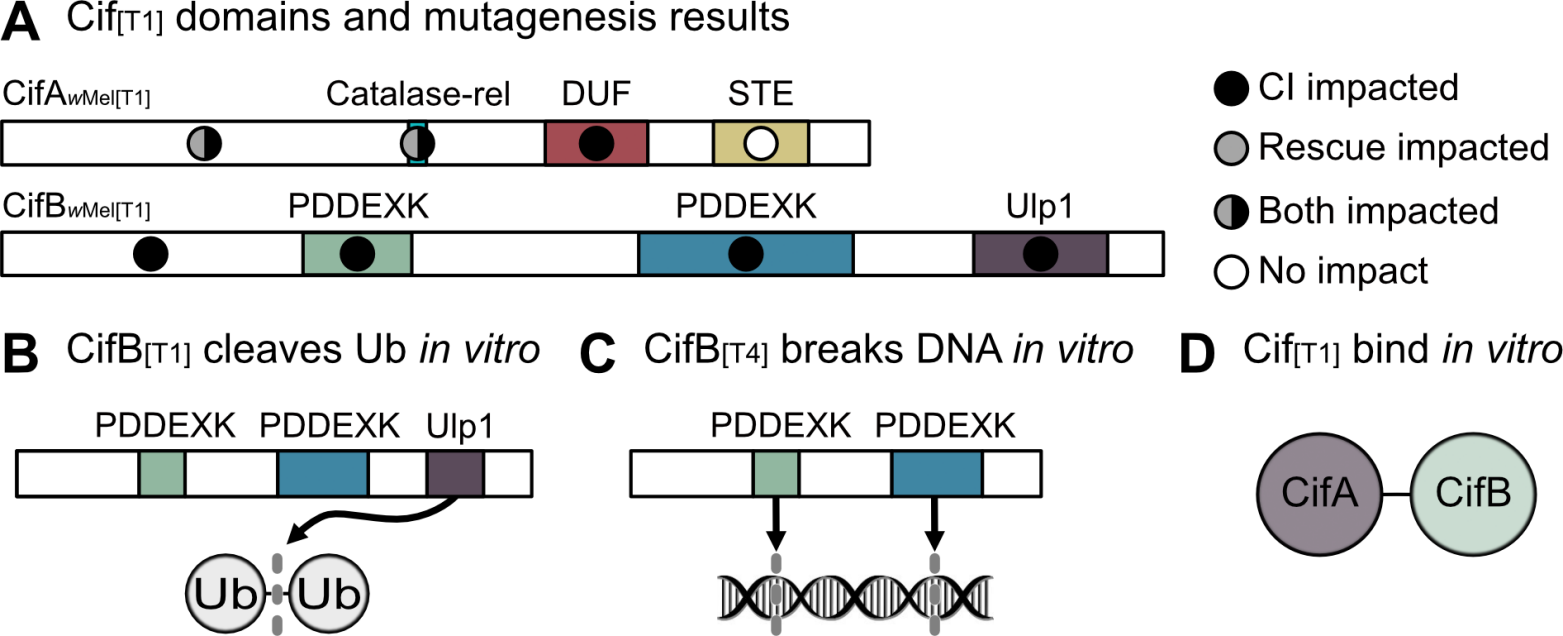
Biochemical characterization of Cif proteins. (**A**) Annotated domains in the CifA and CifB proteins and the relative importance of conserved residues in each domain for CI (black circles), rescue (gray circles) or neither phenotype (white circles) as determined by transgenic expression of mutated proteins in aposymbiotic *D. melanogaster* (Beckmann et al., 2017; Shropshire et al., 2020). (**B**) CifB_[T1]_ can cleave ubiquitin chains via its Ulp1 deubiquitinase domain *in vitro* (Beckmann et al., 2017). (**C**) CifB_[T4]_ nuclease domains can cause DNA breaks *in vitro* (Chen et al., 2019). (**D**) CifA_[T1]_ and CifB_[T1]_ bind each other *in vitro* (Beckmann et al., 2017). Domain architecture is based on homology-based analyses and is of low predictive value (20–30% probability) for CifA (Lindsey et al., 2018), and CifB_[T1]_ PDDEXK nuclease domains lack the canonical PD-(D/E)XK motif (Beckmann et al., 2017), but remain structurally homologous to other PDDEXK nucleases (Lindsey et al., 2018).

Structural homology-based analyses suggest that Type 1 CifA have three putative domains: a catalase-related (catalase-rel) domain involved in the degradation of reactive oxygen species, a domain of unknown function (DUF) 3243 with homology to a Puf-family RNA-binding domain (RBD), and a sterile-like transcriptional regulator (STE) (Figure 4A; Lindsey et al., 2018). While the catalase-rel domain is unique to the CifA_[T1]_, the STE is maintained in Type 1–4 genes (Lindsey et al., 2018), and the Puf-family RBD exists in Type 1–5 genes (Bing et al., 2020b; Martinez et al., 2020). Importantly, these annotations are of low predictive value (20–30% probability) and may not withstand experimental testing (Lindsey et al., 2018). On the other hand, sliding window analyses of selection for CifA_[T1]_ suggest that while the full protein is under purifying selection, the catalase-rel domain and the unannotated N-terminal region are under the strongest selection (Shropshire et al., 2018). Indeed, CifA cannot contribute to transgenic CI or rescue when conserved amino acids are mutated within the unannotated N-terminal region or in the putative catalase-rel domain of CifA_*w*Mel[T1]_ (Shropshire et al., 2020). Conversely, when sites are mutated in CifA’s DUF domain, it maintains the ability to contribute to rescue, but loses CI capability (Shropshire et al., 2020). Thus, CifA’s N-terminal region is crucially important for both CI and rescue, whereas sites within the DUF domain are only crucial for CI (Figure 4A). More work will be necessary to determine how and why these mutations impact these phenotypes, but the annotations provided above afford a set of testable hypotheses and questions. For instance, does CifA interact with reactive oxygen species both in the context of CI and rescue, and/or does RNA-binding occur in the context of CI? Biochemical assays testing for these functions will further elucidate how CifA contributes to CI and rescue phenotypes.

Additionally, how CifA is involved in both CI and rescue remains largely a mystery. The simplest explanation is that CifA maintains the same function in both CI and rescue. Under this framework, CifA would act on a pathway that can be modified during spermatogenesis and oogenesis to produce opposite affects (Shropshire et al., 2018). If CifA were to drive such a function, then CifB’s role in CI would seemingly be auxiliary and perhaps only necessary for localization of CifA to particular targets or, since CifB acts as a deubiquitinase, to protect CifA from degradation by ubiquitin pathways (Beckmann et al., 2017). Alternatively, CifA may be a multi-functional protein that employs one set of functions to cause CI and another to cause rescue (Shropshire et al., 2018). For instance, if CifA targets sex-specific host pathways, CifA can only affect its host in a particular way if that target is available. Additionally, CifA may be modified in some manner that differs between the testes and the ovaries, unlocking unique biochemical functions by posttranslational modification, localization differences, or the expression of different protein conformational isoforms (Shropshire et al., 2018). In summary, little is known about CifA’s functional role in CI and rescue, and considerable work is necessary not only to identify its enzymatic capabilities, but also to further elucidate how it can act both to cause and prevent CI.

### CifB molecular function

CifB_[T1]_ from both *w*Mel and *w*Pip encode a single putative ubiquitin-like protease (Ulp1) domain (Beckmann et al., 2017; LePage et al., 2017; Lindsey et al., 2018). The Ulp1 domain was later expressed separate from the rest of the protein in *E. coli* and purified for downstream *in vitro* ubiquitin cleavage assays (Beckmann et al., 2017). When exposed to a variety of ubiquitin chains, it was revealed that the Ulp1 cleaves K6-, K11-, K27-, K29-, K33-, K48-, and K63-linked ubiquitin *in vitro*, but with a preference for K63 chains (Figure 4B; Beckmann et al., 2017). K63 chains are associated with NF-κB signaling which has diverse functions including innate immunity, DNA transcription, autophagocytosis (Tan et al., 2008; Wertz and Dixit, 2010), and proliferation of cell nuclear antigen (PCNA) (Ripley et al., 2020) that has previously been shown to act abnormally in CI-affected embryos (Landmann et al., 2009). A single amino acid mutation in the catalytic site of the Ulp1 prevents the breakdown of ubiquitin chains *in vitro* (Beckmann et al., 2017). Expressing the Ulp1 catalytic mutant for CifB*_w_*_Pip[T1]_ and CifB*_w_*_Mel[T1]_ in male *D. melanogaster* alongside CifA did not induce CI, suggesting that deubiquitilase activity is important for CI barring the occurrence of any potential protein structural changes in the mutants (Figure 4A; Beckmann et al., 2017; Shropshire et al., 2020). However, some caution is warranted as deubiquitinase assays have not been conducted using the full-length protein, and it is unknown if this activity is maintained *in vivo*. Moreover, it remains unknown what CifB deubiquitinates, if anything, inside reproductive tissue cells and how this deubiquitination contributes to CI. It is also interesting that nuclear localization of the male-killing protein Spaid in the endosymbiont *Spiroplasma poulsonii* is impacted by a domain annotated as a deubiquitinase, suggesting that host ubiquitin regulation or localization to host nuclei is important for reproductive manipulation (Harumoto and Lemaitre, 2018). Future biochemical assays will help answer these persistent questions.

While CifB’s Ulp1 is seemingly important for CI, only CifB_[T1]_ have this domain. Moreover, additional mutagenesis assays reveal that other conserved sites across the CifB protein similarly ablate CI function (Figure 4A; Shropshire et al., 2020), strongly suggesting other regions of the protein are likewise important for CifB function. For instance, all CifB proteins (Type 1–5) are also annotated with a dimer of PD-(D/E)XK (hereafter PDDEXK) nuclease domains (Bing et al., 2020b; Lindsey et al., 2018; Martinez et al., 2020). Indeed, *in vitro* nuclease assays with CifB*_w_*_Pip[T4]_ confirm that they can nick both double- and single-stranded DNA (Figure 4C; Chen et al., 2019). Moreover, mutating PDDEXK catalytic sites in CifB*_w_*_Pip[T4]_ prevents nuclease activity *in vitro* and CI-inducibility when expressed in *D. melanogaster* (Chen et al., 2019). Unlike the other phylogenetic Types, CifB_[T1]_ proteins do not have the canonical PDDEXK catalytic sites, thus lending doubt to the importance of these domains as nucleases in CifB_[T1]_ (Beckmann et al., 2017). However, these domains remain structurally homologous to other PDDEXK domains (Lindsey et al., 2018), and many functional PDDEXK-like domains lack the canonical PD-(D/E)XK catalytic motifs, opting instead for alternative catalytic residues and structural folds. PDDEXK-like domains without catalytic sites are still involved in other DNA-associated processes (Knizewski et al., 2007). Mutating conserved amino acid residues in either of the PDDEXK domains of CifB*_w_*_Mel[T1]_ inhibits its ability to contribute to CI (Shropshire et al., 2020). Additionally, despite *w*Pip containing both Type 1 and 4 genes, there are no notable differences in cytological embryonic defects caused when both genes are expressed as compared to other strains that only have CifB_[T1]_, suggesting that these genes yield similar cytological outcomes (Bonneau et al., 2018b). Biochemical assays will be necessary to evaluate the nuclease activity of a diverse array of CifB proteins including CifB_[T1]_ because the conserved areas in and around the PDDEXK domains across all Cif Types likely persist because of a common function that underpins CifB’s involvement in CI.

### Cif interacting partners

A list of putative Cif protein-binding partners have recently been reported. While CifA_[TI]_ and CifB_[T1]_ bind *in vitro* (Figure 4D; Beckmann et al., 2017), it does, however, remain unknown if CifA binds CifB *in vivo* in the testes to cause CI or if maternal CifA binds to paternal CifB in the embryo to cause rescue (Beckmann et al., 2019a; Beckmann et al., 2019b; Shropshire et al., 2019). More work on the localization, co-localization, and binding profiles of these proteins will elucidate this question. Additionally, Cifs appear to bind to a suite of host proteins that differ based on if CifA and CifB are expressed alone or together. Sixty-seven host proteins were identified as Cif binding partners under co-expression of CifA and CifB, whereas 45 proteins were identified with CifB expression alone in pools of male and female *D. melanogaster* (Beckmann et al., 2019c). Karyopherin-α (Kap-α) is notable among these proteins. It bound to singly expressing CifB extracts, and its overexpression in aposymbiotic females yielded partial rescue (~20% hatch rate improvement) when crossed to transgenic CI males (Beckmann et al., 2019c). Kap-α is a nuclear import receptor and a regulator of p53 which has roles in the protamine-histone exchange process (Beckmann et al., 2019c; Emelyanov et al., 2014). Intriguingly, delayed H3 histone deposition is a hallmark of CI during early embryogenesis (Landmann et al., 2009), suggesting a relationship between CifB, Kap-α, p53 and histone-associated abnormalities in CI. However, it is important to emphasize that Kap-α was only pulled down when CifB was singly expressed (Beckmann et al., 2019c), suggesting that while Kap-α overexpression may influence rescue-efficiency, it is unclear how it would be part of the rescue mechanism since CifB is not necessary for rescue to occur. More work is essential to determine if CifB’s binding to Kap-α contributes to CI and how Cif binding to other host proteins relates to CI and rescue phenotypes.

### What is the cytological basis of CI?

**Figure 5.**
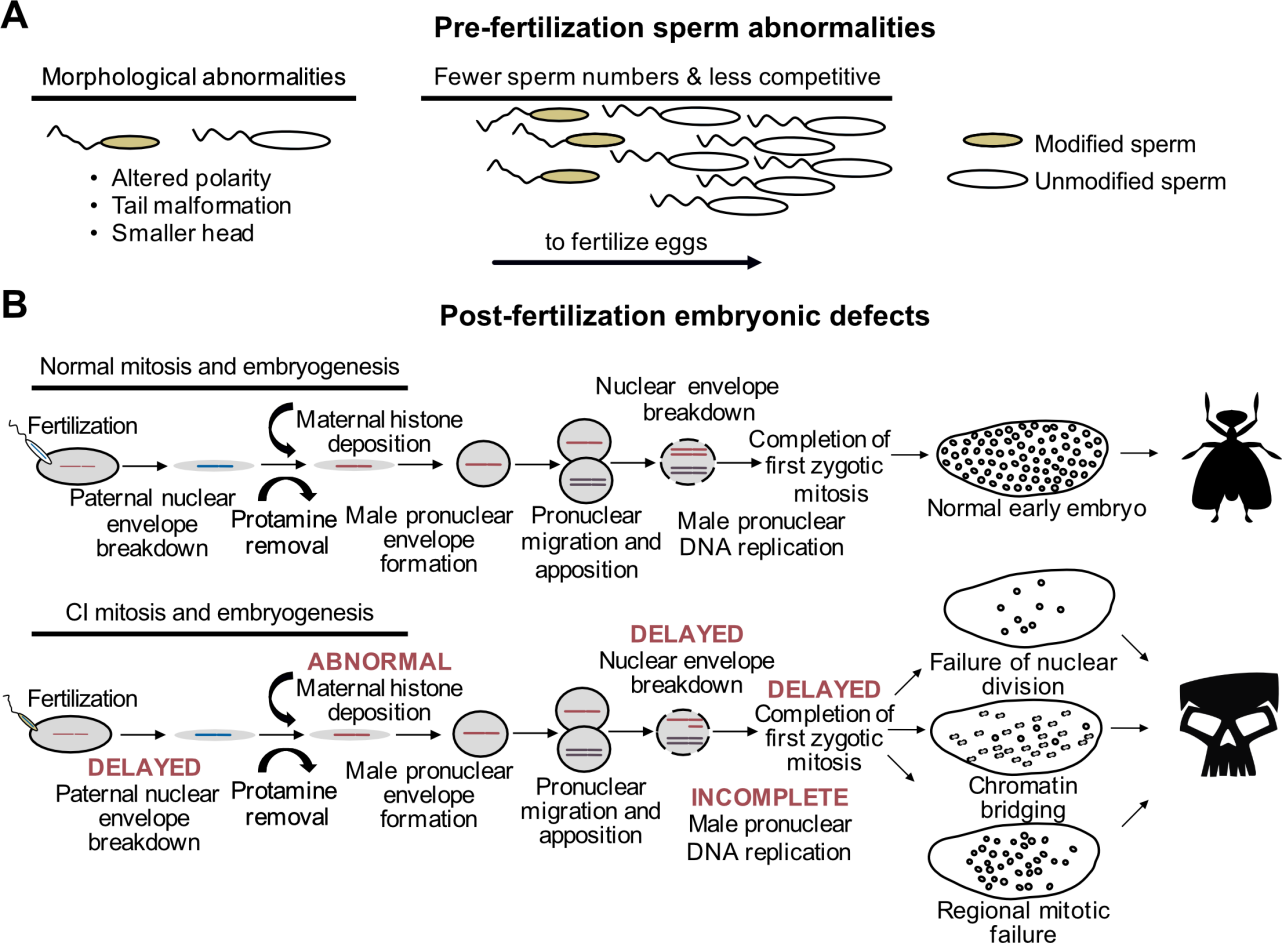
CI-associated defects occur pre- and post-fertilization. (**A**) In males harboring *Wolbachia*, there are several types of sperm abnormalities when compared to their aposymbiotic counterparts. (**B**) When fertilized with sperm derived from *Wolbachia*-carrying males, embryonic nuclear defects result in the form of delayed paternal nuclear envelope breakdown, abnormal histone deposition and other early mitotic events. These defects then cause embryonic phenotypes observed in CI including chromatin bridging and regional mitotic failures.

Decades of research have characterized an in-depth understanding of CI-associated cytological abnormalities. These studies broadly define alterations during spermatogenesis (Figure 5A) and embryogenesis (Figure 5B), suggesting that CI is associated with a sperm modification prior to fertilization that results in embryonic defects and death. Importantly, the causes of the reported sperm and embryonic abnormalities remain unknown and, in most studies, it is unclear if these observations are directly related to the Cif proteins or are a byproduct of *Wolbachia* symbionts in the testes. However, these findings provide insight into the ways in which *Wolbachia* and CI influence host reproduction and fertility. Below, we review the cytological changes that occur in spermatogenesis and embryogenesis during CI and rescue, and we highlight areas where future research is crucially needed with reductionist assays to disentangle effects of *Wolbachia* symbiosis and CI.

### CI-associated abnormalities prior to fertilization

Spermatogenesis is a highly regulated process. It begins with cells of the germline stem cell niche (GSCN) replicating into spermatogonia that subsequently undergo mitosis to yield a spermatocyst with 16 spermatocytes (Fuller, 1993; Hackstein, 1987; Lindsley, 1980). Each spermatocyte in the cyst then undergoes two rounds of meiosis to form four spermatids, for a total of 64 spermatids in each cyst. The spermatids then undergo elongation where the sperm tail forms, and histones are replaced with protamines for tight packaging of DNA in the nucleus within the sperm head (Rathke et al., 2014). In the final stages of sperm maturation, spermatids undergo individualization to remove excess cytoplasm before becoming mature sperm to enter the seminal vesicle for storage before mating. Impacts of *Wolbachia* on spermatogenesis can result in downstream sperm defects that may be connected to CI (Figure 5A). For example, symbiont-bearing *D. simulans* flies and *Ephestia* moths produced fewer sperm, and stronger CI was associated with more sperm transfer during copulation in *D. simulans* (Awrahman et al., 2014; Lewis et al., 2011; Snook et al., 2000). When *D. simulans* females mated with *Wolbachia*-bearing and aposymbiotic males, the sperm of aposymbiotic males were more likely to fertilize eggs (Champion de Crespigny and Wedell, 2006), suggesting that *Wolbachia*-modified sperm are less competitive. *Wolbachia*-affected sperm cysts exhibit abnormal morphology with some sperm fused together and other sperm exhibiting randomly-oriented, axoneme-mitochondrial complexes that are responsible for sperm motility (Riparbelli et al., 2007), perhaps explaining fertility defects and variation in sperm competition. However, key questions remain. Are *cif* gene products responsible for the aforementioned sperm abnormalities? If these defects are not caused by the *cif* genes, and are instead a byproduct of other *Wolbachia*-host associations, what is their significance, if any, to reproductive parasitism? In summary, a deeper investigation of these defects as they relate to CI products is needed to confirm their link to CI.

Interestingly, *Wolbachia* are not symmetrically distributed in testes, with only some spermatocysts harboring symbionts in the strong CI-inducing *w*Ri strain of *D. simulans* (Clark et al., 2003). Indeed, *w*Ri is almost exclusively localized to the GSCN, and some GSCN remain aposymbiotic, suggesting that the Cif proteins must either act early in spermatogenesis or are diffusible factors that can stably travel into later stages of spermatogenesis (Clark et al., 2003; Clark et al., 2002; Riparbelli et al., 2007). *Wolbachia* are stripped during the individuation process and moved into waste bags where they are presumably degraded (Riparbelli et al., 2007). Not only does this suggest that *Wolbachia* create a diffusible factor that interacts with sperm or spermatogonia to cause CI, but it also helps to explain why paternal *Wolbachia* transfer has not been observed (Yeap et al., 2016) with rare exceptions such as in hybrid *Nasonia* wasps and in transinfected *A. aegypti* (Chafee et al., 2011; Ross et al., 2020a). Future work investigating the localization of CifA and CifB will determine when and where the Cif proteins act to cause CI and whether they are transferred to the embryo for the potential to directly cause defects during embryogenesis.

### CI-defining abnormalities after fertilization

Abnormalities that define the post-fertilization events underpinning CI are observed during embryogenesis (Figure 5B). In chronological order, CI-affected embryos experience abnormal maternal H3.3 histone deposition on the male pronucleus, delayed activation of the DNA polymerase cofactors PCNA and cell cycle regulator Cdk1 resulting in incomplete DNA replication, delayed nuclear envelope breakdown prior to the first mitosis, and a delay in the first mitotic event (Landmann et al., 2009; Tram and Sullivan, 2002). These defects often culminate in a chromatin bridging phenotype during the first mitosis, shredding the paternal nuclei and leading to embryonic arrest (Breeuwer and Werren, 1990; Callaini et al., 1996; Lassy and Karr, 1996; Reed and Werren, 1995; Ryan and Saul, 1968; Tram et al., 2006). Notably, it remains unknown what the most proximal event is during CI. It is plausible that abnormal histone deposition is the first CI-causing event during embryogenesis that leads to a cascade of effects culminating in the other embryonic abnormalities, but there remain open questions. For instance, how do the Cif proteins interact with the host to cause abnormal histone deposition? Are the Cif proteins even transferred with the sperm so that they can cause these defects directly, or are these defects caused by an initial Cif interaction occurring during spermatogenesis? Finally, if Cifs do directly cause abnormal histone deposition, how are these affects rescued by the presence of CifA expressed in the embryo? A combination of cytological, transgenic, and biochemical assays may be necessary to evaluate these questions.

Defects in the first mitotic division are traditionally viewed as a key cytological outcome of CI, but abnormalities later in embryogenesis are also common and increasingly appreciated (Bonneau et al., 2018b; Callaini et al., 1997; LePage et al., 2017; Ryan and Saul, 1968). There are three distinct phenotypes: early mitotic failures whereby embryonic arrest occurs after several successful rounds of division, regional mitotic failures where some regions of the embryo appear to be dividing without issue, and widespread chromatin bridging in later stages of division (LePage et al., 2017). The cause of these defects remains unknown, but at least two hypotheses can be proposed. First, late stage embryonic defects are caused by the same cascade of abnormalities that often cause arrest during the first mitosis. Under this scenario, the difference in the cytological outcomes of the embryo may be explained by the magnitude of the proximal CI-causing affect. For instance, strong male pronuclear delay can result in complete exclusion of the male pronucleus from early development, yielding an embryo that may attempt to undergo haploid development (Callaini et al., 1997; Tram et al., 2006). In *N. vitripennis* where haploid individuals become males and diploids become females, exclusion of the male pronucleus during CI can manifest in haploidization where even fertilized eggs develop as haploids (Bordenstein et al., 2003; Ryan and Saul, 1968; Tram et al., 2006; Vavre et al., 2001; Vavre et al., 2000). Thus, the intensity of pronuclear delay may correspond with the resulting phenotypic profile during embryogenesis, but more work is necessary to determine if these effects translate to late stage embryonic defects. Second, these later stage abnormalities may be independent from the defects preceding the first mitotic failure. Indeed, it has been proposed that different phylogenetic Types of Cif proteins may contribute to different cytological outcomes (Bonneau et al., 2018b). However, *w*Pip, which encodes both Type 1 and 4 genes, display both early and late stage embryonic abnormalities comparable to *w*Mel which encodes only Type 1 genes (Bonneau et al., 2018b; LePage et al., 2017). Despite presumably having different mechanistic bases, *Cardinium* and *Wolbachia* both have converged on similar outcomes during early embryogenesis, including chromatin bridging and abnormal number of chromosomes after the first division (Gebiola et al., 2017). It remains unknown if *Cardinium* CI yields comparable molecular defects and sperm abnormalities to *Wolbachia*-induced CI. Clearly, there are a diverse set of cytological outcomes associated with CI in both *Wolbachia* and *Cardinium*. Additional cytological and reductionistic studies will be necessary to evaluate the cause of this variation and determine how the Cif proteins contribute to these phenotypes.

## What is the host’s contribution to CI?

It is common that researchers leverage correlations between *Wolbachia* symbiont state and host expression phenotypes (RNA, protein, etc.) to understand how *Wolbachia* impact their host. When differential expression is correlated with CI phenotypes, these data can yield valuable insights regarding CI’s mechanism. Significant correlations between *Wolbachia* symbiont state and host expression have been measured in *D. melanogaster* (Biwot et al., 2020; He et al., 2019; LePage et al., 2014; Liu et al., 2014; Ote et al., 2016; Xi et al., 2008; Yuan et al., 2015; Zheng et al., 2019b; Zheng et al., 2011), *D. simulans* (Brennan et al., 2012; Clark et al., 2006; Xi et al., 2008), *La. striatellus* (Huang et al., 2019; Ju et al., 2017; Liu et al., 2019), *T. urticae* (Bing et al., 2020a; Zhang et al., 2015)*, Cu. pipiens* (Pinto et al., 2013), and *A. albopictus* (Baldridge et al., 2017; Baldridge et al., 2014; Brennan et al., 2012; Brennan et al., 2008). Challengingly, as many as 1613 transcripts are differentially expressed between *Wolbachia* symbiont states (Bing et al., 2020a), and as with the cytological abnormalities described above, it is difficult to untangle the effects of *Wolbachia* and CI on host expression profiles.

However, the most promising candidates associated with CI are those that can be experimentally over- or under-expressed to recapitulate CI-like hatch rates and cytological defects. For example, overexpression of the tumor suppressor gene *lethal giant larvae* [l(2)gl] and myosin II gene *zipper* in aposymbiotic *D. simulans* induces a considerable reduction in hatching that is accompanied with CI-associated cytological defects (Clark et al., 2006). However, CI is not just associated with hatch rate defects, but also the ability to rescue those defects. When l(2)gl and *zipper* over-expressing males were mated to symbiont-bearing females, no change in hatching was observed (Clark et al., 2006), suggesting that hatch rate reductions associated with these factors cannot be rescued and thus are not CI-associated. Nevertheless, there have been numerous studies that have identified host factors that contribute to CI-like embryonic abnormalities and can be rescued by symbiont-bearing females: the aminotransferase *iLve* which mediated branched-chain amino acid biosynthesis in *La. striatellus* (Ju et al., 2017), the sRNA nov-miR-12 which negatively regulates the DNA-binding protein *pipsqueak* (*psq*) in chromatin remodeling in *D. melanogaster* (Zheng et al., 2019b), cytosol amino-peptidase-like which are in the sperm acrosome and involved in fertilization in *La. striatellus* (Huang et al., 2019), two seminal fluid proteins (CG9334 and CG2668) with unknown function in *D. melanogaster* (Yuan et al., 2015), the histone chaperone *Hira* in *D. melanogaster* and *D. simulans* (Zheng et al., 2011), a Juvenile Hormone protein (JHI-26) involved in development in *D. melanogaster* (Liu et al., 2014), and the immunity-related gene *kenny* (*key*) in *D. melanogaster* (Biwot et al., 2020). Since misexpression of these host products in aposymbiotic males mimic CI-like embryonic defects in a way that can be rescued by symbiont-bearing females, there is support that these products or their pathways are involved in CI, but how these factors relate to cause CI remains unknown, and there is no current evidence that these are binding partners with Cif proteins.

In addition to RNA and/or protein expression differences, changes in host physiology and cell biology are correlated with CI. For example, *Wolbachia*-bearing *D. melanogaster, D. simulans*, *A. albopictus*, *A. polynesiensis*, and *T. urticae* males often have higher reactive oxygen species (ROS) in their testes than aposymbiotic males (Brennan et al., 2012; Brennan et al., 2008; Zug and Hammerstein, 2015). It has been hypothesized that this variation in ROS expression patterns is due to an elevated host immune response to *Wolbachia* symbiosis (Zug and Hammerstein, 2015). However, multiple lines of evidence link ROS expression with CI. For example, increased ROS levels are consistently observed among CI-inducing strains (Zug and Hammerstein, 2015), and ROS leads to DNA damage in spermatocytes in *D. simulans* (Brennan et al., 2012). Additionally, lipid hydroperoxide markers of ROS-induced oxidative damage are higher in symbiont-bearing *D. melanogaster* (Driver et al., 2004), and PCNA retention is another marker for DNA damage and is observed during the first mitosis of CI-affected embryos (Landmann et al., 2009). Interestingly, overexpression of the *D. melanogaster* gene *key* increases ROS levels and DNA damage in males when mimicking rescuable CI-like hatching and embryonic defects (Biwot et al., 2020). Together, these data support a role for ROS in CI’s mechanism, but direct connections remain unclear. One hypothesis is that CifA’s putative catalase-related domain does indeed function to interact with ROS (Lindsey et al., 2018). Though, alternatively, ROS may be a byproduct of the host immune response (Zug and Hammerstein, 2015). Biochemical and immunological assays will unravel these relationships.

## What causes variation in CI strength?

**Figure 6.**
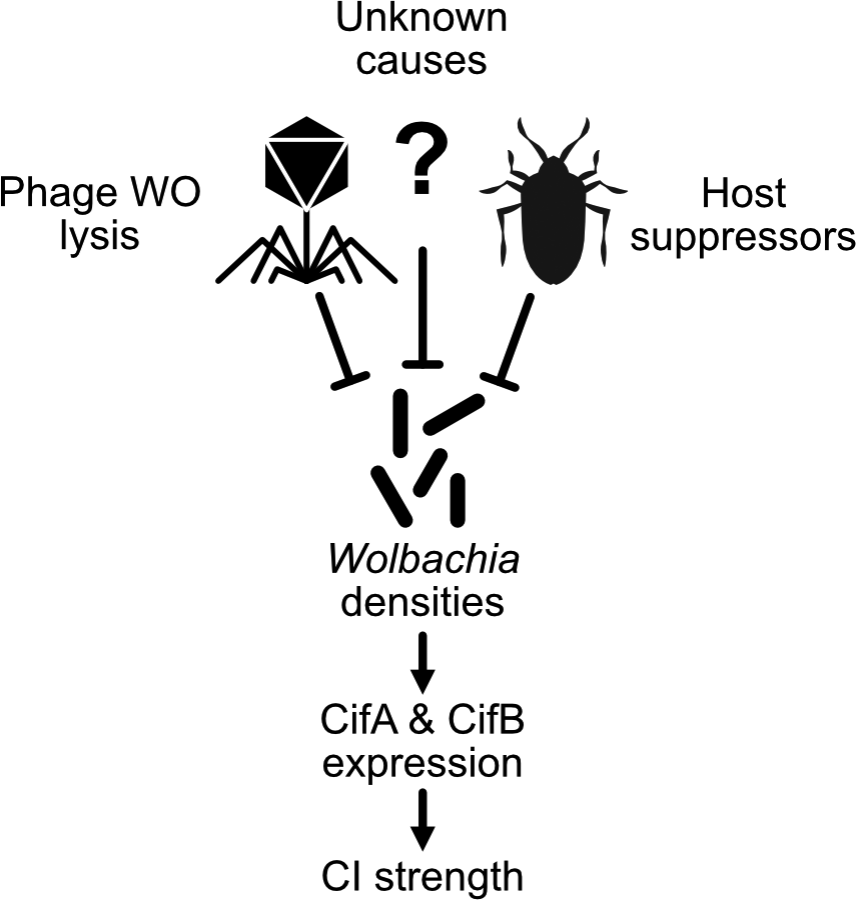
An expanding *Wolbachia* density model of CI strength variation. The proximal cause of CI is likely CifA and CifB, whose transcriptional level has been connected with intensity in transgenic studies (LePage et al., 2017). *Wolbachia* densities have often correlated with factors that influence CI strength variation (Werren, 1997). In many cases, it remains unknown how these factors influence *Wolbachia* densities. Phage WO lysis (Bordenstein and Bordenstein, 2011) and host suppressors are well documented correlates or causes of density changes (Funkhouser-Jones et al., 2018; Poinsot et al., 1998; Walker et al., 2011).

Some *Wolbachia* strains exhibit CI that can vary between 10–100% embryonic death (Awrahman et al., 2014; Clark et al., 2003; Cooper et al., 2017; Hoffmann, 1988; Layton et al., 2019; Reynolds and Hoffmann, 2002; Turelli et al., 2018; Yamada et al., 2007; Zabalou et al., 2004). In fact, a number of *Wolbachia,* including *w*Mel of *D. melanogaster* and *w*Yak of *D. yakuba*, were initially characterized as non-parasitic since they had minimal to no impact on embryonic hatching (Charlat et al., 2004; Holden et al., 1993; Zabalou et al., 2004). Later studies would correct these early reports to suggest they can indeed cause CI, but their CI strength is highly dependent on a variety of factors including the age of fathers and grandmothers (Cooper et al., 2017; Layton et al., 2019; Reynolds and Hoffmann, 2002). Generally speaking, the work reviewed below describes a complex relationship between biotic and abiotic factors that influence CI strength. Notably, the bacterial density model of CI, whereby *Wolbachia* densities positively correlate with CI strength, is likely the major factor driving most of these relationships (Breeuwer and Werren, 1993). Phage WO lysis, host suppressors, and other undescribed interactors may control the variation in *Wolbachia* titers (Figure 6; Awrahman et al., 2014; Bordenstein and Bordenstein, 2011; Funkhouser-Jones et al., 2018; Layton et al., 2019; Poinsot et al., 1998; Walker et al., 2011). However, there are instances where CI strength variation does not correlate with *Wolbachia* densities (Yamada et al., 2007). Below, we review these works and describe what is known and unknown about the proximal basis of CI strength variation.

### Temperature

Temperature is often correlated with CI strength and is likely to contribute to the dynamics that govern *Wolbachia*’s spread (Foo et al., 2019). High temperatures, usually exceeding 27°C, can have a significant negative impact on CI strength in *Wolbachia*-carrying *A. aegypti* (Ross et al., 2020b; Ross et al., 2019), *T. urticae* (van Opijnen and Breeuwer, 1999), *D. simulans* (Hoffmann et al., 1986), *D. melanogaster* (Reynolds and Hoffmann, 2002), *A. scutellaris* (Trpis et al., 1981; Wright and Wang, 1980), *A. albopictus* (Wiwatanaratanabutr and Kittayapong, 2009), and *Nasonia* (Bordenstein and Bordenstein, 2011). There is considerable evidence that high temperature impacts *Wolbachia* densities in various species including *A. albopictus* and *A. aegypti* (Foo et al., 2019; Ross et al., 2020b; Ross et al., 2019), *N. vitripennis* (Bordenstein and Bordenstein, 2011), and *T. urticae* (Lu et al., 2012). High temperatures even cure hosts *of Wolbachia* (Jia et al., 2009). In natural populations of the butterfly *Zizeeria maha*, *Wolbachia* densities vary with season, and climate change may be contributing to a decrease in symbiont frequencies in the tropics (Charlesworth et al., 2019; Sumi et al., 2017). Notably in *N. vitripennis* and *T. urticae*, decreased *Wolbachia* densities and CI strength have also been correlated with an increase in phage WO lytic activity with higher temperatures (Bordenstein and Bordenstein, 2011; Lu et al., 2012). Cooler temperatures at or below 19°C have also been associated with decreased CI in *D. simulans* and *N. vitripennis* (Bordenstein and Bordenstein, 2011; Reynolds and Hoffmann, 2002). As with warm temperatures, cooler temperatures also yield increased phage WO densities, decreased *Wolbachia* densities, and decreased CI strength in *N. vitripennis* (Bordenstein and Bordenstein, 2011). These data suggest a phage density model of CI wherein phage WO may respond to temperature extremes by increasing its replication and lysing bacterial cells, thus lowering overall *Wolbachia* densities and resultantly CI levels.

However, while robust support of this model is available in *N. vitripennis* (Bordenstein and Bordenstein, 2011), more work is necessary to test if it is generalizable to other *Wolbachia* strains. For instance, in contrast to the relationships described above, *Wolbachia* in some *D. simulans* lines (Clancy and Hoffmann, 1998) and *Leptopilina heterotoma* wasps (Mouton et al., 2006) replicate more quickly at warmer temperatures, and yet CI strength decreases. Thus, it is plausible that phage WO in these species have a different relationship with temperature than in *N. vitripennis*, and other yet undescribed factors inhibit CI. Moreover, in *E. suzannae* bearing *Ca. hertigii*, high temperatures also yield reduced *Cardinium* densities and lower CI strength (Doremus et al., 2019). However, *Cardinium* do not harbor a phage, and thus phage lysis cannot explain this relationship. Additionally, in this same system, cooler temperatures yield reduced *Cardinium* densities, but an increase in CI strength (Doremus et al., 2019). Thus, here, it seems that bacterial densities alone do not explain the cause of CI strength variation. It is plausible that the factors contributing to CI strength variation in *Wolbachia* and *Cardinium* differ, and comparative phenotypic studies will be necessary to evaluate the differences between these two systems. However, in systems where symbiont density correlates with CI strength, it is plausible that the proximal cause is a shift in CI gene expression that correlates with symbiont densities. Transcript and protein abundance assays of *Wolbachia*’s *cif* genes will help elucidate this relationship when accompanied with measurements of variable CI strength.

### Host behavior and development

Other correlates of CI strength variation are related to male and paternal grandmother age (Awrahman et al., 2014; Layton et al., 2019; Reynolds and Hoffmann, 2002), male mating rate (Awrahman et al., 2014; de Crespigny et al., 2006), male developmental timing (Yamada et al., 2007), rearing density (Yamada et al., 2007), and nutrition (Clancy and Hoffmann, 1998). All of these factors are significantly impacted by the structure of the population, resource availability, or behavior. Below, we will systematically discuss what, if anything, is known about how each of these factors impact CI strength.

First, male age can be negatively correlated with CI strength. For example, *w*Mel of *D. melanogaster* has nearly no impact on embryonic hatching when males are 3–5 days of age, but can induce significant CI when males are less than 2 days of age (Reynolds and Hoffmann, 2002). Similar results have been observed with *Wolbachia* in *D. simulans* and *N. vitripennis*, but to varying degrees (Breeuwer and Werren, 1993; Karr et al., 1998). Since *Wolbachia* densities decrease with male age in *D. melanogaster, D. simulans,* and *N. vitripennis* hosts (Binnington and Hoffmann, 1989; Breeuwer and Werren, 1993; Bressac and Rousset, 1993; Clark et al., 2002; Karr et al., 1998; Reynolds and Hoffmann, 2002; Riparbelli et al., 2007; Turelli and Hoffmann, 1995; Veneti et al., 2003; Weeks et al., 2007), it is perhaps unsurprising that age also correlates with CI. Moreover, of the factors associated with CI strength, age is also the only one that has been investigated in the context of *cifA* and *cifB* transcription, and does indeed decrease with age alongside *Wolbachia* densities (LePage et al., 2017).

Interestingly, while older males have fewer *Wolbachia*, older virgin females have more (Layton et al., 2019). In fact, when females are aged longer prior to mating, their male offspring are laid with higher *Wolbachia* densities and resultantly induce stronger CI (Layton et al., 2019). This phenomena has been termed the paternal grandmother age effect (PGAE) (Layton et al., 2019). It is unclear why age’s impact on *Wolbachia* density is sex-specific. However, the relationship between male age, symbiont densities, and CI strength may not be generalizable across all CI-inducing symbionts and their hosts. For instance, *Cardinium* of *E. pergandiella* cause CI that is unaffected by male age (Perlman et al., 2014), and studies disagree about the significance of the impact of age on CI caused by *w*Ri of *D. simulans* (Awrahman et al., 2014; Binnington and Hoffmann, 1989; Bressac and Rousset, 1993). Thus, the impacts of age on symbiont densities and CI may be limited to some *Wolbachia* or alternatively, to particular host backgrounds. More comparative phenotypic work will be needed to understand the broader context of the relationship between age, CI strength, and *cif* expression.

Additionally, male mating rate is also negatively correlated with CI strength. For instance, symbiont-bearing *D. simulans* males mate more frequently than aposymbiotic males, and the increased mating rate yields weaker CI in later matings (Awrahman et al., 2014; de Crespigny et al., 2006). Symbiont-bearing males also transfer more sperm during copulation than aposymbiotic males during the first mating encounter, and decreased sperm transfer in subsequent matings corresponds with weaker CI (Awrahman et al., 2014). As such, the increased mating frequency may be a behavioral adaptation employed by some hosts to restore reproductive compatibility between symbiont-bearing males and aposymbiotic females (Awrahman et al., 2014). As with temperature and age (Bordenstein and Bordenstein, 2011; Layton et al., 2019; Reynolds and Hoffmann, 2002), it has likewise been hypothesized that *Wolbachia* densities may decrease upon remating (Awrahman et al., 2014), but this hypothesis has not been tested. Alternatively, it has also been hypothesized that the amount of time that sperm remains in contact with *Wolbachia* corresponds with how strong CI can be (Karr et al., 1998), thus remating may contribute to high sperm turnover that limits *Wolbachia*-sperm exposure. These hypotheses can be tested via *Wolbachia* density assays and microscopy of reproductive tissues upon remating.

Male development time is likewise correlated with CI strength. Here, *w*Mel-bearing male *D. melanogaster* induce stronger CI when they are the first emerging males of a clutch (Yamada et al., 2007). The younger brothers, which take longer to develop but are approximately the same age, cause weaker CI. This phenotype has been termed the younger brother effect (YBE) (Yamada et al., 2007). The YBE is an outlier in phenotypes associated with CI strength variation in that younger and older brothers have comparable adult *Wolbachia* densities (Yamada et al., 2007), suggesting an alternative mechanism for the relationship between developmental timing and CI strength. However, it is also plausible that while younger and older brothers have similar bacterial densities, their localization may shift such that cells more important to CI expression have higher densities than other cells in the testes (Clark et al., 2002). Alternatively, *Wolbachia* densities of the adult male may be less informative than density differences during embryonic or larval development. For instance, the PGAE, as described above, revealed that sons of older females caused stronger CI and while their sons did not have higher *Wolbachia* densities as adults, they did have higher densities during embryogenesis (Layton et al., 2019). Intriguingly, *Wolbachia* densities rapidly declined in aged females after mating and embryo laying, suggesting that many *Wolbachia* were transferred from the ovaries to the developing egg and ultimately embryo. Thus, it is plausible that *Wolbachia* densities would correlate with deposition order such that first laid older brothers would have higher densities than younger brothers laid soon after (Layton et al., 2019). While these hypotheses remain associated and to be formally tested, it is also notable that the YBE does not appear to apply to *w*Ri of *D. simulans* (Yamada et al., 2007), other studies have failed to replicate these results in other *w*Mel-bearing *D. melanogaster* lines (LePage et al., 2017), and the opposite phenotype is observed with *Cardinium* of *Encarsia* where older brothers cause weaker CI (Perlman et al., 2014). Thus, additional work is necessary to replicate the YBE in *D. melanogaster* and other symbiont-host combinations and to evaluate its cause via longitudinal developmental studies of *Wolbachia* densities. Moreover, understanding why *Cardinium* and *Wolbachia* CI are differentially impacted by these factors is important in determining how symbiont dynamics relate to reproductive manipulation.

Finally, rearing density and nutrition can also impact CI strength relationships. For instance, when *w*Mel-bearing *D. melanogaster* are reared in high densities, CI strength is lower than if they are reared in low densities (Yamada et al., 2007). The initial hypothesis behind this correlation was that high-density rearing led to nutritional stress which translated to less *Wolbachia* (Yamada et al., 2007). While this hypothesis has not been explicitly tested, there is a reasonable logical framework behind it. Indeed, multiple studies have shown that *D. simulans* males exposed to nutritional stress have weaker CI than males with abundant resources (Clancy and Hoffmann, 1998; Sinkins et al., 1995). Notably, nutritional stress is also correlated with reduced *Wolbachia* densities, supporting models, as above, where *Wolbachia* densities and *cif* expression are the proximal factors driving the relationship to CI strength. That said, it is important to note that recent microscopy studies have shown that standard qPCR-based measures of *Wolbachia* densities may not be adequate under nutritional stress since host ploidy is subject to variation based on diet (Christensen et al., 2019). Thus, it is plausible that qPCR-based variation in *Wolbachia* densities under nutritional stress may in fact be driven by variation in host ploidy and not *Wolbachia* density. Replication of these studies will be necessary to confidently link nutrition, rearing density, and *Wolbachia* densities to CI strength. Importantly, rearing density does not influence CI strength in *w*AlbA and *w*AlbB *A. albopictus* (Dutton and Sinkins, 2004), suggesting that even if *w*Mel CI is impacted by rearing-density, this effect is perhaps not generalizable across *Wolbachia*-host combinations.

The factors described above do not work on CI in isolation but instead seem to be mingled in a state of perpetual complexity. For instance, the impact of temperature on CI strength in *D. melanogaster* is dependent on male age, where 1-day-old males reared at 25°C induce stronger CI than those reared at 19°C, but the inverse is true with 3- and 5-day-old males (Reynolds and Hoffmann, 2002). Moreover, age has a variable impact on CI strength in different host backgrounds, suggesting that genotypic variation in either the host or *Wolbachia* strain may impact these relationships (Duron et al., 2007a; Reynolds and Hoffmann, 2002). These studies highlight the complexity of *Wolbachia*-host-environment interactions and should motivate additional investigation to resolve the factors that underpin these variations and the host genetic loci that influence how impactful each factor might be in each host.

### Host genetics

Relationships between *Wolbachia* phenotypes and host genotypes are frequently investigated through transinfections of a *Wolbachia* strain into a non-native background via injection (Hughes and Rasgon, 2014) or introgression of one species cytoplasm into another host’s background via repeated backcrossing (Chafee et al., 2011). For example, *w*Mel *Wolbachia* of *D. melanogaster* traditionally cause weak CI (Holden et al., 1993), but induce consistently strong CI when transinfected into either *D. simulans* or *A. aegypti* (Poinsot et al., 1998; Walker et al., 2011). Similar results were also observed when *w*Tei which induce weak or no CI in the *D. yakuba* complex (Charlat et al., 2004; Cooper et al., 2017; Martinez et al., 2020; Zabalou et al., 2004), are transferred into *D. simulans* (Zabalou et al., 2008). Moreover, despite seemingly carrying the same *Wolbachia*, different genetic lineages of the wasp *N. longicornis* express different compatibility relationships with other strains (Raychoudhury and Werren, 2012), and *w*VitA of *N. vitripennis* causes weak CI in its native host but strong CI when introgressed into *N. giraulti* (Chafee et al., 2011). Intriguingly, this affect is only observed with *w*VitA and does not apply to *w*VitB, which also causes CI, suggesting that both host and *Wolbachia* genetics play a role in CI phenotypes. These studies support models that predict hosts will be selected to develop resistance against CI (Prout, 1994; Turelli, 1994), and raise many questions about an evolutionary arms race between *Wolbachia* and its host to control reproductive parasitism.

There are at least two broad models for the mechanisms of host suppression of CI: divergence of host products that are targets for CI (defensive model) or evolution of host products that inhibit and suppress *Wolbachia* or CI products (offensive model). First, a defensive model would predict that the pathway(s) that CI act(s) on in the host must be conserved enough for CI to be transferable between species, but also malleable enough for the pathway(s) to become resistant to CI. The host genes, transcripts, and proteins described earlier in this review are excellent candidates since they can mimic CI phenotypes (Biwot et al., 2020; Huang et al., 2019; Ju et al., 2017; Yuan et al., 2015; Zheng et al., 2011). However, studies are necessary to investigate genetic variation in these host products to assess the possibility that they are under selection to suppress CI. Other candidates would be direct binding partners of CifA and CifB (Beckmann et al., 2019c), but nothing is known about how natural variation in these products may relate to CI suppression. Conversely, an offensive model could yield the evolution of host genes involved in *Wolbachia* density regulation or some other target with indirect effects on CI strength. Notably, since these products may not be involved in the CI mechanism, they would not necessarily be expected to be conserved. For instance, the *Wolbachia* density suppressor (*Wds*) gene of *Nasonia* acts to suppress densities of *w*VitA, is taxon restricted to bees and wasps, and is under positive selection as would be expected for a suppressor acting in an evolutionary arms race with *Wolbachia* (Funkhouser-Jones et al., 2018). Since *Wds* is only present in Hymenoptera, it clearly is not generalizable as a standard mechanism of *Wolbachia* and CI suppression. However, it is plausible that other hosts have converged on comparable mechanisms of CI suppression. Additional research will be needed to reveal the diversity of mechanisms surrounding CI suppression and to understand the dynamics controlling their evolution.

## What are the models for *Wolbachia*-induced CI and rescue?

**Figure 7.**
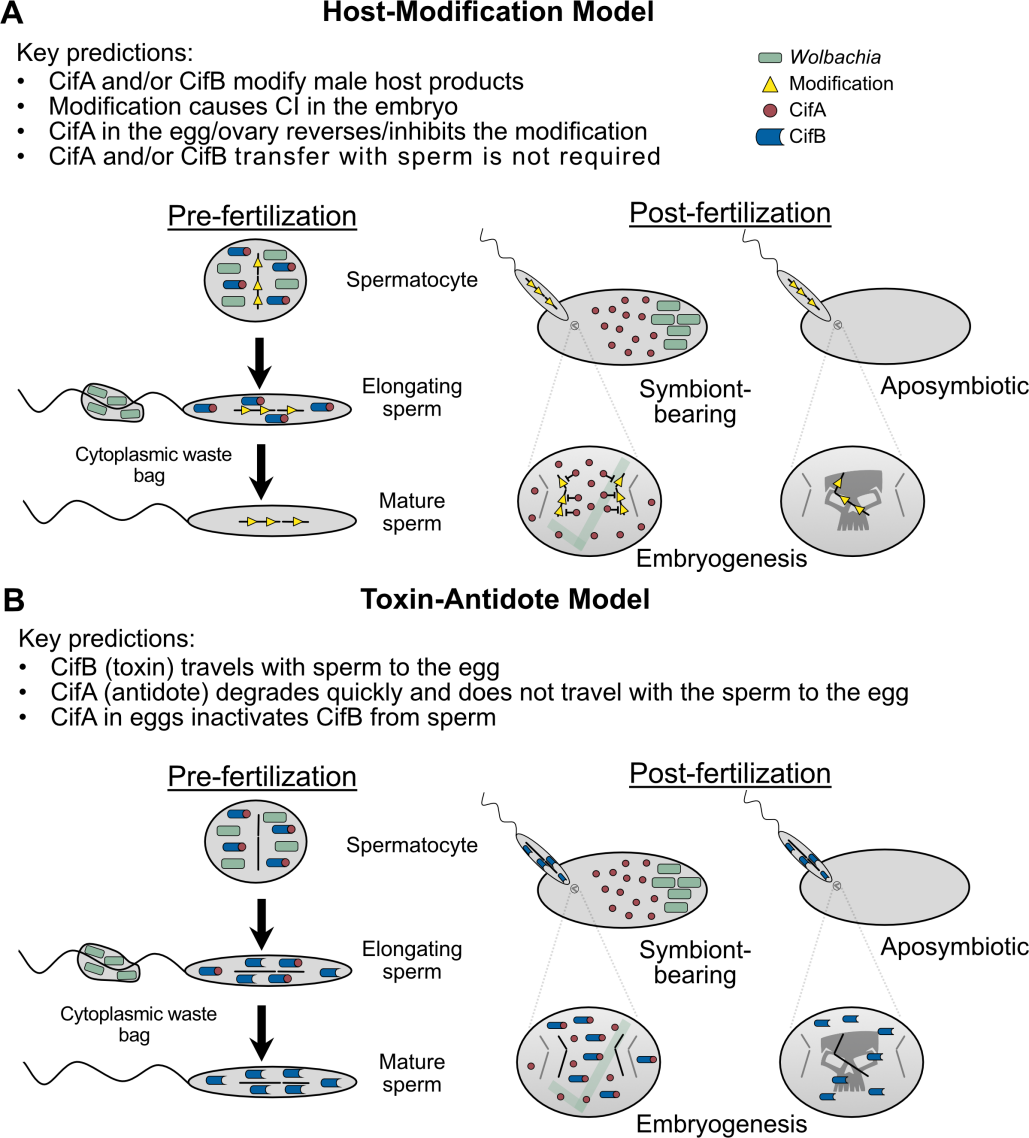
The Host-Modification and Toxin-Antidote models of CI mechanism. (**A**) The Host-Modification (HM) model predicts that the Cif proteins impart a modification on male-derived products that result in CI unless CifA is available in the embryo to reverse or otherwise inhibit the male-derived modification (Shropshire et al., 2019; Werren, 1997). (**B**) The Toxin-Antidote (TA) model predicts that CifB is the primary toxin that is transferred to the embryo via the sperm, and that rescue occurs when CifA binds CifB in the embryo and inhibits its toxicity (Beckmann et al., 2019a; Hurst, 1991; Shropshire et al., 2019; Werren, 1997).

Numerous models have been proposed to explain CI and rescue mechanisms. First, we discuss the utility of the classical phenotype-based modification/rescue (*mod*/*resc*) model in a post-genomic world (Shropshire and Bordenstein, 2019; Werren, 1997). Additionally, despite considerable advances in the genetics and biochemistry of CI, numerous mechanistic models are used to describe CI and rescue (Beckmann et al., 2019a; Poinsot et al., 2003; Shropshire et al., 2019). These models divide into two discrete categories: host-modification (HM) (Figure 7A) and toxin-antidote (TA) (Figure 7B; Beckmann et al., 2019a; Shropshire et al., 2019). HM-based models assume that the CI-inducing factors act directly to modify host male products and that rescue occurs through either removal of these modifications or otherwise reversing the effects through a separate host-modification in the female. TA-based models assume that the CI-inducing factors are transported into the embryo via the sperm and are toxic after fertilization unless the rescue factor is present, binds to the CI toxin, and inhibits its toxicity. We discuss each of these models and their variants in the context of a Two-by-One genetic framework (Shropshire and Bordenstein, 2019), but it is crucial to reemphasize that while both CifA and CifB proteins are required to induce CI, the specific biochemical mechanism underlying each protein’s contribution to CI and rescue remains unknown and there remains insufficient data to confirm any of these models.

### The mod/resc model

The *mod*/*resc* model defines a *mod* factor as a CI-inducing product produced in males and a *resc* factor as a rescue-inducing product produced in females (Werren, 1997). The *mod*/*resc* model is agnostic to the genetic, biochemical, enzymatic, or cytological basis of CI. Instead, the *mod*/*resc* model provides a framework for describing the phenotypic expression of different *Wolbachia* strains. For example, a standard CI-inducing strain that can self-rescue would be denoted as *mod*+/*resc*+. Less common phenotypes include so-called suicidal *Wolbachia* (*mod*+/*resc*-) and *Wolbachia* that do not cause CI but can rescue CI induced by other strains (*mod*-/*resc*+) (Ant and Sinkins, 2018; Meany et al., 2019; Zabalou et al., 2008). *Wolbachia* that do not cause CI or rescue are designated *mod*-/*resc*-.

The *mod*/*resc* model assumes that for bidirectional CI to occur, the *mod* and *resc* factors would differ in such a way that they remain functional but are incompatible with each other (Charlat et al., 2001; Werren, 1997). As such, a strain can carry multiple *mod* or *resc* factors that determine the compatibility relationships with other strains, and the *mod*/*resc* model can be used to estimate the number of *mod* and *resc* factors within a host (Zabalou et al., 2008). To do this, *Wolbachia* strains are transinfected or introgressed into the same genetic background and then crossed to determine the incompatibility relationships between strains or against aposymbiotic flies. A strain that causes CI against an aposymbiotic female is considered to have at least one mod factor. If it can rescue itself then it has at least one *resc* factor. If two CI-inducing and self-compatible strains are bidirectionally incompatible, then it is assumed that each carry at least one set of *mod* and *resc* factors but that they are not the same. Indeed, crossing experiments between various *Wolbachia* strains have revealed unidirectional and bidirectional incompatibilities which have led to agreement that *Wolbachia* frequently carry multiple *mod* and *resc* factors (Poinsot et al., 1998; Zabalou et al., 2008).

With the identification of the CI and rescue genes (Beckmann et al., 2017; Chen et al., 2019; LePage et al., 2017; Shropshire et al., 2018; Shropshire and Bordenstein, 2019), it is compelling to abandon the *mod*/*resc* model in favor of a purely genetic description of CI relationships. With the ever-growing availability of genomic datasets, acceptance of a gene-centric analysis of CI may be the simplest way to predict CI of a symbiont. However, while sequence information can indeed yield informed hypotheses about a strain’s CI, some hosts suppress their symbiont’s CI (Chafee et al., 2011; Poinsot et al., 1998; Walker et al., 2011), and some symbiont strains exhibit different forms of reproductive parasitism based on their host background (Fujii et al., 2001; Jaenike, 2007; Sakamoto et al., 2005; Sasaki et al., 2002; Zabalou et al., 2008). Thus, we propose that a modern framework for describing CI relationships should involve both phenotypic data described under the *mod*/*resc* model and genetic data described under the Two-by-One model. For example, if genomic sequencing of a novel *Wolbachia* precedes phenotypic observations, then a genetic analysis could reveal *cifA* and *cifB* homologs that are either comparable to those in CI-inducing strains or contain putative loss-of-function mutations. Phenotypic data is of course necessary to confirm the hypothesis. Indeed, *w*Yak of *D. yakuba* and *w*Rec of *D. recens* cause CI, but they have *cifB* genes with stop codons that truncate the proteins relative to the *w*Mel and *w*Pip *cifB* (Cooper et al., 2017; Martinez et al., 2020; Shoemaker et al., 1999). As such, a genetic description of these strains alone could result in mischaracterization of *w*Yak and *w*Rec as non-parasitic strains with putative *cifB* pseudogenes. It is only with knowledge of both *cif* gene sequence and phenotypic data that a complete understanding of the basis of CI in these strains is possible. Thus, both the Two-by-One and *mod*/*resc* models will serve as a useful framework to describe these systems.

### HM-based mechanistic models

HM models (Figure 7A) make at least two key predictions. First, male host products are modified by Cifs (Shropshire et al., 2019). There are numerous, pre-fertilization defects associated with CI-inducing *Wolbachia* including changes in sperm morphology and competitive ability (Champion de Crespigny and Wedell, 2006; Riparbelli et al., 2007), supporting that the host is modified prior to fertilization. It is unknown, however, whether these outcomes are due to pre-fertilization defects causally related to CI or general responses to *Wolbachia* in the testes. Second, and most crucially, the proximal CI modifications causing death of the fertilized embryo is rescued (e.g., replaced or otherwise negated) by CifA in the embryo (Shropshire et al., 2018; Shropshire and Bordenstein, 2019). CifA does not rescue through binding with male-transferred CifB products since Cif protein yields the modification prior to fertilization in testes and thus does not need to be transfered with the sperm to the embryo. Instead, CifA may interact with host processes to reverse or otherwise stop the effects of CI caused by CifA and CifB protein expression in males. As such, assessment of the location of CifA and CifB binding (testes or embryo), the transfer of Cif products, if any, with the sperm, and the interactions that Cif have with the host will further inform this model. We discuss three additional non-exclusive HM-based models below: titration-restitution, mistiming, and goalkeeper.

**Figure 8.**
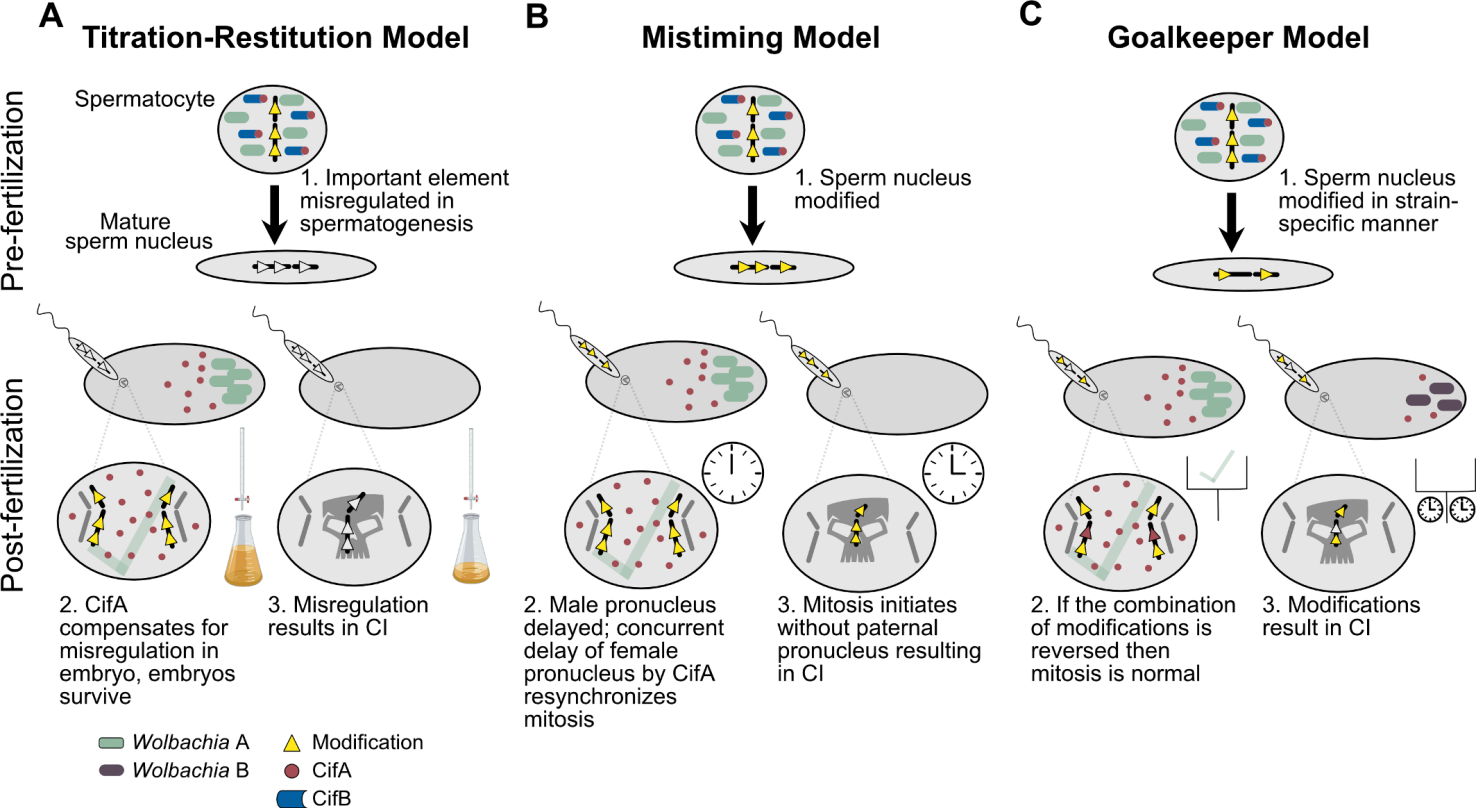
Extensions of the Host-Modification model. (**A**) The Titration-Restitution Model posits that an element within mature sperm is either over- or under-expressed in males due to Cif protein expression, but this alteration is then remedied in the female as a result of CifA through a reconstitution of the required element (Werren, 1997). (**B**) The Mistiming Model posits that a modification in the male sperm causes a delay in the formation of the male pronucleus that results in CI if CifA does not cause a concurrent delay in the maternal pronucleus, resynchronizing mitosis between the two pronuclei (Tram and Sullivan, 2002). (**C**) The Goalkeeper Model expands on the Mistiming Model and posits that the male product modification occurs in a strain-specific quantity, and may involve multiple modifications that need to be remedied to rescue the lethality (Bossan et al., 2011).

The titration-restitution model (a.k.a. the ‘sink’ hypothesis) was originally proposed by Werren, 1997 and posits that CI is induced by over- or under-expression of host products or pathways in the testes/sperm and rescue occurs when the same products are misregulated in the opposite direction in the ovaries/embryo (Figure 8A; Kose and Karr, 1995; Poinsot et al., 2003; Werren, 1997). Indeed, *Wolbachia* have a considerable impact on expression profiles, some host genes are differentially expressed in male and female reproductive tissues, and numerous host factors meet these criteria (Baldridge et al., 2017; Baldridge et al., 2014; Bing et al., 2020a; Yuan et al., 2015), as described in the section above. There are at least two ways in which CifA and CifB proteins can underpin the titration-restitution model. First, since CI and rescue would occur through titration of the same host product or pathway, it is feasible that CifA, which acts on both sides of the phenotype (Shropshire and Bordenstein, 2019), may drive these expression changes. Under such a model, CifB may act as an ‘accessory protein’ that enables CifA to target a paternally derived product that it would otherwise not be able to reach on its own. Second, CifA may act on its own to up- or down-regulate host products but has the opposite impact on that product when CifB is present. As such, rescue would occur through CifA’s lone action which counteracts the misregulation caused by CifA and CifB dual expression.

Notably, titration-restitution models can explain bidirectional CI if Cif products from different strains have variable impacts on multiple host expression pathways. Thus, rescue would not be possible from a second strain since it could be targeting the wrong host factor or pathway. Indeed, divergent CI genes may differentially impact host pathways. For example, only the CifB_[T1]_ sequences maintain a functional Ulp1 domain while the other four CifB clades have a dimer of PDDEXK nucleases that is also present in CifB_[T1]_ (Beckmann et al., 2017; Bing et al., 2020b; Lindsey et al., 2018; Martinez et al., 2020). It is feasible that CifB with different domains impact different host pathways. Alternatively, Cif proteins may have differential impacts on the level of misregulation instead of or in addition to impacting multiple host pathways which may influence incompatibility relationships. More work will be necessary to understand if *cif* expression influences transcriptional and translational variation and how that variation corresponds to CI.

The mistiming model (a.k.a. the ‘slow motion’ hypothesis) was first explicitly proposed by Tram and Sullivan, 2002 and is based on the observation that the paternal pronucleus has slowed development relative to the female pronucleus in CI crosses, the rescue cross has normal cell cycle timing, and the female pronucleus continues development despite the slowdown in both *Drosophila* and *Nasonia* species (Figure 8B; Callaini et al., 1996; Ferree and Sullivan, 2006; Ryan and Saul, 1968; Tram and Sullivan, 2002). This established the hypotheses that delayed male pronuclear development is responsible for emergent defects in early embryogenesis, and that resynchronization of the development may occur by comparably slowing down the development of the female pronucleus or slowing the cell cycle in rescue. Since the cell cycle timing of the female pronucleus is what establishes the timing for the first mitosis (Bossan et al., 2011), symbiont-bearing females do not induce CI because the male pronucleus reach apposition prior to the female pronucleus. Though, the reciprocal cross would be incompatible because the female pronucleus finished development prior to the male, and the first mitosis would have initiated before the male pronucleus arrives. Importantly, this model predicts that CI crosses are subject to haploidization of diploid offspring since the male pronucleus could be completely excluded from mitosis if it was significantly slowed. This is indeed the case in *N. vitripennis* where CI often manifests as only male offspring since haploid offspring are viable in this species but develop as males (Bordenstein et al., 2003).

The mistiming model proposes that CI and rescue have comparable impacts on the development of male and female gametes, respectively. As such, a single gene could in theory be responsible for both CI and rescue (Poinsot et al., 2003). Under this paradigm, CifA may enact a slowdown in both tissues since it is involved in both phenotypes (Shropshire and Bordenstein, 2019). However, if this were the case, then what would be the purpose of CifB? It is possible that CifB is responsible for localizing CifA to a male-specific target where it imposes the same outcomes on its host. Since this hypothetical male product would not be available in the embryo, CifB would not have a role in rescue. However, an alternative model for mistiming is that rescue may not occur through slowing down the female pronucleus but may instead work by removing the slowdown agents from the male pronucleus. Together, these models would help to explain the proximal cause (misregulation) and culminating effects (mistiming) of CI. More work will be necessary to understand if rescue occurs via slowdown of the female pronucleus or from speeding-up the male pronucleus.

A major limitation of the mistiming model is that it cannot explain bidirectional CI. Since mistiming proposes that rescue happens through delaying the female pronucleus as much as or greater than the male pronucleus, a sufficiently strong delay should yield compatibility with any strain that has a weaker male delay. As such, only unidirectional CI should manifest between strains where the strain inducing the stronger delay is capable of rescue. The goalkeeper model was proposed in 2011 as a way to address this limitation (Figure 8C; Bossan et al., 2011). In addition to the expectations of the mistiming model, goalkeeper suggests that a secondary factor unassociated with this mistiming may also be involved in CI. The combined contributions of these two mod factors leads to CI. Under this paradigm, CifA and CifB may contribute to different kinds of defects during spermatogenesis, each contributing in somewhat independent ways to CI. Rescue must then negate the impacts of both factors. Thus for CifA to rescue CI, it would not only need to contribute to a delay in the pronuclear development but also reverse the impacts of a secondary source of modification. Notably, since the titration-restitution model does not make predictions about the developmental timing of the male and female pronuclei, it is compatible with both mistiming and goalkeeper models and can help explain mistiming through misregulation of host factors in a manner that leads to slowed development. More functional genetic, biochemical, and cytological studies are necessary to understand how a goalkeeper model and/or a combination of these HM-based models may contribute to CI.

### TA-based mechanistic models

Since *Wolbachia* are not paternally inherited, Hurst proposed in 1991 that *Wolbachia* make a CI-inducing toxin that diffuses into the sperm cytoplasm and is transferred to the egg during fertilization and causes death (Hurst, 1991). Rescue then occurs when *Wolbachia* in the egg produce an antidote that binds to the toxin and prevents it from killing the embryo (Hurst, 1991). This TA model (Figure 7B) makes two key predictions (Beckmann et al., 2019a; Shropshire et al., 2019). First, the Cif proteins are transferred to the embryo. Mass spectrometry of spermatheca from symbiont-bearing *Cu. pipiens* females mated with symbiont-bearing males revealed fragments of CifA (Beckmann and Fallon, 2013). These later data have been used to support this prediction, but since these females harbor *Wolbachia* (Beckmann and Fallon, 2013) and CifA is also the rescue protein (Chen et al., 2019; Shropshire et al., 2018), the most parsimonious explanation for CifA’s presence in symbiont-bearing spermatheca is related to *Wolbachia* in females and potentially rescue, not CI. It remains possible that Cif proteins are transferred, but this is not the simplest interpretation of currently available data. Second, if the proteins are transferred, then maternal CifA must bind to the CI toxin to prevent function. *In vitro* biochemical assays reveal that CifA and CifB are capable of binding (Beckmann et al., 2017), but it remains unknown if they bind as a toxin complex to induce CI or if CifA binds to CifB in the embryo to rescue CI. Moreover, while CifB’s Ulp1 domain is an *in vitro* deubiquitinase, CifA’s binding to CifB does not inhibit deubiquitinase activity, suggesting that if binding is for the purpose of rescue it is not inhibiting one of CifB’s biochemical functions (Beckmann et al., 2017). As such, assays investigating if the Cif products are transferred to the embryo at all and where the Cif proteins bind each other in reproductive tissue cells will inform the foundation of this hypothesis.

The TA model traditionally states that the toxin and antidote are separate factors (Poinsot et al., 2003). However, our genetic understanding is that CifA is involved in both CI and rescue. There are two ways to update the model to be consistent with a Two-by-One genetic framework (Shropshire and Bordenstein, 2019) while maintaining the key assumptions of the TA model (Hurst, 1991). First, CifB may be the sole toxin but requires CifA as an antidote even during spermatogenesis to prevent overly defective sperm (Beckmann et al., 2019a). For this to work, CifA is expected to degrade faster than CifB, leaving CifB alone to enter the egg as a toxin unless it binds to maternally-derived CifA (Beckmann et al., 2019a). Alternatively, CifA and CifB could work together as a toxin complex that enters the embryo and is then rescued by maternallyA. Binding assays coupled with microscopy and localization studies will reveal when and where CifA acts relative to CifB.

As described above, the TA model aims to explain unidirectional CI between symbiont-bearing and aposymbiotic individuals. A modification of the TA model, called lock-and-key, expands the TA model to explain incompatibilities between *Wolbachia* strains. The lock-and-key model, like TA, proposes that a toxin is transferred from symbiont-bearing males to the embryo and will cause embryonic death unless an antidote is supplied. Toxins in this case are called locks, and antidotes are keys. The toxin lock is proposed to bind to or otherwise interfere with factors associated with proper embryonic development unless the antidote key is available to remove the lock. Bidirectional CI can then be explained by one strain carrying a set of locks and keys that are not compatible with the other strains’ locks and keys because of differences in binding affinity. This model leveraged predictions of the *mod*/*resc* model that strains can have multiple sets of *mod*/lock and *resc*/key factors and that a key is more likely to bind to its associated lock than to a divergent lock. Indeed, *Wolbachia* exhibit considerable *cif* polymorphism (Bing et al., 2020b; Bonneau et al., 2019; LePage et al., 2017; Lindsey et al., 2018; Martinez et al., 2020) and binding of CifA and CifB is strongest between cognate partners (Beckmann et al., 2017). However, the lingering questions with the TA model also apply with the lock-and-key model. Additionally, validation that divergent Cif proteins are functional, that they have differential impacts on the host, and contribute summatively to incompatibilities are lacking.

## Conclusion

*Wolbachia* were first discovered in *Cu. pipiens* mosquitoes in 1924 and later linked to CI in 1973 (Hertig and Wolbach, 1924; Yen and Barr, 1973). Since then, advances have significantly expanded our recognition of *Wolbachia*’s incredible and complex toolset. In particular, biologists now appreciate CI as a common form of reproductive parasitism that symbionts, including *Wolbachia* and *Cardinium*, use to rapidly spread through populations (Hunter et al., 2003; Rosenwald et al., 2020; Takano et al., 2017; Turelli, 1994; Weinert et al., 2015; Zug and Hammerstein, 2012). CI is associated with reproductive isolation (Bordenstein et al., 2001; Gebiola et al., 2017; Jaenike et al., 2006) and is leveraged as a successful tool in the prevention of arboviral diseases that infect humans (Crawford et al., 2020; O'Neill, 2018; Tantowijoyo et al., 2020). The last decade has seen a rapid expansion in our understanding of phage WO’s role in CI genetics (Beckmann et al., 2019c; Chen et al., 2019; LePage et al., 2017; Shropshire et al., 2018; Shropshire and Bordenstein, 2019), phylogenetics (Bing et al., 2020b; LePage et al., 2017; Lindsey et al., 2018; Martinez et al., 2020), and mechanism (Beckmann et al., 2019c; Beckmann et al., 2017; Chen et al., 2019; Shropshire et al., 2020). Moreover, considerable effort has been made to describe CI-defining cytological defects (Ferree and Sullivan, 2006; Landmann et al., 2009), link variation in host expression with CI phenotypes (Biwot et al., 2020; Liu et al., 2014; Zheng et al., 2011), and untangle factors that influence CI strength such as *Wolbachia* densities and phage WO lytic activity (Bordenstein and Bordenstein, 2011; Layton et al., 2019; Reynolds and Hoffmann, 2002; Yamada et al., 2007). Together, this significant body of literature has motivated models to explain how CI works (Beckmann et al., 2019a; Bossan et al., 2011; Poinsot et al., 2003; Shropshire et al., 2019). These studies have stone-by-stone erected a steady foundation that will serve as a launching point for exciting new discoveries to fully appreciate the complexity of this powerful form of reproductive manipulation. Looking forward, key areas of investigation will involve the relative roles of CifA and CifB in the induction of CI, the cell biology of the Cif proteins, the genetic basis of bidirectional CI, the cytogenetic basis of CI strength variation, linkage of Cif expression with cytological abnormalities pre- and post-fertilization, Cif-induced CI’s molecular and biochemical basis, and mechanisms of host suppression of CI.
